# Toward a better modulus at shallow indentations—Enhanced tip and sample characterization for quantitative atomic force microscopy

**DOI:** 10.1002/jemt.24261

**Published:** 2022-11-18

**Authors:** David S. Owen

**Affiliations:** ^1^ Department of Physics and Astronomy University of Sheffield Sheffield South Yorkshire UK

**Keywords:** contact mechanics, polydimethylsiloxane, probe geometry, quantitative atomic force microscopy, scanning electron microscopy

## Abstract

Approximations of the geometry of indenting probes, particularly when using shallow indentations on soft materials, can lead to the erroneous reporting of mechanical data in atomic force microscopy (AFM). Scanning electron microscopy (SEM) identified a marked change in geometry toward the tip apex where the conical probe assumes a near linear flat‐punch geometry. Polydimethylsiloxane (PDMS) is a ubiquitous elastomer within the materials and biological sciences. Its elastic modulus is widely characterized but the data are dispersed and can display orders of magnitude disparity. Herein, we compare the moduli gathered from a range of analytical techniques and relate these to the molecular architecture identified with AFM. We present a simple method that considers sub‐100 nm indentations of PDMS using the Hertz and Sneddon contact mechanics models, and how this could be used to improve the output of shallow indentations on similarly soft materials, such as polymers or cells.

## Graphical abstract

Assessment of the geometry of indenting probes at the tip apex is crucial during shallow indentations—as is an understanding of the interaction between the probe and the material of interest. This study demonstrates a simple method to calibrate the atomic force microscopy (AFM) to extract quantitative moduli of soft cells and materials.

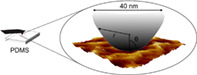

**Research Highlights**

- High‐resolution scanning electron microscopy (SEM) of the tip apex of an atomic force microscopy (AFM) probe shows a marked change in geometry from the prescribed nominal values.
- Utilized these geometries within the Hertz model of contact mechanics to enable more accurate and quantitative elastic moduli of soft materials and polymers, such as Polydimethylsiloxane (PDMS).
- Comparisons of the molecular architecture of PDMS at the surface and sub‐surface.

## 1 INTRODUCTION

Attempts to define the contact angles on rough surfaces can be problematic as the relationship between the intrinsic contact angle and the apparent contact angle may vary from point to point (Wolansky & Marmur, 1999). Solid surfaces contain irregular deviations from their prescribed geometry at the sub‐micron level. These are typically characterized by peaks (asperities) or low points (valleys). When two nominally flat surfaces are brought together the surface roughness leads to contact at discrete points along their contacting regions. These discrete points constitute the real contact area, and it is usually only a small proportion of the expected contact area, had the surfaces been perfectly smooth (Bhushan, 1998). Several different mathematical models of contact mechanics seek to address these factors, each with their own strengths and limitations. The Hertz (Hertz, 1882), Sneddon (Sneddon, 1948), Johnson–Kendal–Roberts (JKR; Johnson et al., 1971), or Derjaguin–Muller–Toporov (DMT; Derjaguin et al., 1975) models are widely used for the assessment of soft materials. The merits and limitations of each are hotly contested in the literature, and such debates are beyond the scope of this paper. For the researcher with little experience in contact mechanics, or those who may be restricted to a software embedded model, their options may be limited. We have utilized the Hertz and Sneddon models, which are widely used in the material (Denisin & Pruitt, 2016; Domke & Radmacher, 1998; Lim & Chaudhri, 2004; Mesarovic & Fleck, 1999) and biomechanical sciences (Chopinet et al., 2013; Eaton et al., 2008; Smolyakov et al., 2016a; Solopova et al., 2016). These models do not consider the adhesive contribution, and are best suited when adhesion is negligible—such as when analysis is performed in an aqueous environment (Suriano et al., 2014). Despite this limitation, many studies of soft materials are published each year using the Hertz model. Moreover, even when competing models show differences in elastic modulus on the same sample they are typically small, and reside within the same order of magnitude (Suriano et al., 2014). Incorrectly identifying the indenter radius can lead to differences of elastic moduli spanning many orders of magnitude within the same sample. With adequate insight into the correct geometries of an indenting probe and by minimizing the influence of adhesion—particularly if analyzing in an aqueous environment, it is possible to extract reliable, quantitative data using the Hertz model.

The shape at the end of the tip is critical when indenting to depths much smaller than the indenter radius (Cohen & Kalfon‐cohen, 2013). During instrumented nanoindentation (INI), the experimenter makes great attempts to fully characterize the geometry of the indenter. However, the atomic force microscopy (AFM) community do not appear to be as zealous. Often, the manufacturer designated nominal tip radii or conical half‐angles are used in the contact mechanics models routinely employed. Alternatively, blind reconstruction of the tip geometry may be utilized (Bailey et al., 2014b; Cohen & Kalfon‐cohen, 2013). This technique has proved useful to identify changes in tip geometry which can occur over the course of repeated scans on hard substrates, and transmission electron microscopy (TEM) can provide high resolution micrographs of these changes (Liu et al., 2010; Vahdat et al., 2013). Equally, many of the published reports using scanning electron microscopy (SEM) also relate to the wear and damage to the tip following repeated scans on a hard surface (Park et al., 2014; Xue et al., 2014). However, the resolution of these images does not always allow for detailed radii or angle measurements near the tip apex. Moreover, tip wear and changes in geometry should be negligible when scanning on soft substrates, such as those used in this study.

Polydimethylsiloxane (PDMS) is an organosilicon elastomer, widely used in the life sciences. It is extensively used in microfluidic devices (Fujii, 2002; Johnston et al., 2014; Tsai et al., 2011) as it has shown biocompatibility with a diverse range of biomolecules (Bélanger et al., 2001). Its soft and elastic nature allows for reversible deformations and it can be lithographically molded with high fidelity (Jahed et al., 2017; Liu et al., 2009a). Being optically transparent to wavelengths from the infrared to the ultraviolet (Liu et al., 2009b) it displays little autofluorescence (Piruska et al., 2005), thus rendering it as a useful substrate for optical microscopy. Tensile loading or INI are widely used to characterize the elastic modulus of PDMS. In tensile loading the sample is stretched uniaxially and the stress–strain curve is analyzed to derive the modulus. INI applies a compressive, calibrated force, and the accurate determination of the contact point is then used to define the depth of indentation. However, for soft materials, the initial contact point is difficult to determine, which can lead to erroneous reporting of indentation depth and modulus (Cohen & Kalfon‐cohen, 2013). It has been suggested that the modulus increases at increased depths, due to a greater contact of the polymer and the indenter (De Paoli & Volinsky, 2015) and others suggest the opposite—that the modulus decreases during increased indentations, with higher moduli at the surface. This has been attributed to a spherical indenter geometry having a large contact area at the surface (Charitidis, 2011), a dependence of the film thickness (Liu et al., 2009a), or as a result of the molecular properties of PDMS—where it is suggested that there is a greater crosslinking density between the surface and down to 300 nm, whereby the bulk properties change after this depth (Charitidis & Koumoulos, 2012). INI typically operates within a range of hundreds of nanonewtons (nN) to low micronewtons (μN) and is routinely used to determine the mechanical properties of very stiff materials, at micrometer depths. If very small indentations, on the order of tens of nanometers (nm), on a soft substrate are required, INI may not be an ideal choice. AFM is more suited for the characterization of soft materials as it permits the application of smaller loading forces (Celik et al., 2009). Here, a calibrated displacement is applied to the probe and its deflection is typically measured optically (Meyer & Amer, 1988). This interaction between the probe and the sample generates a force–distance (*F*–*D*) curve that contains a variety of quantitative data. The *F*–*D* curve is then usually analyzed by fitting against a contact mechanical model.

Long‐chain polymers typically exist in disordered random coils. The polymeric PDMS has a molecular chain width of around 0.7 nm and the disordered coil should be around 10 nm in thickness (Granick et al., 2003; Yamada, 2003). From an imaging standpoint these structural details are yet to be elucidated with sufficient resolution. Super‐resolution microscopy has investigated PDMS microchannels within a fluidic device (Cheng et al., 2013) and SEM and AFM have observed the surface of native and coated PDMS (Davis et al., 2021; Liamas et al., 2021; Nourmohammadi et al., 2015; Yu et al., 2013), which showed considerable porosity. In this work we have characterized the molecular architecture of PDMS at the surface and sub‐surface respectively and obtained high‐resolution electron micrographs of a commonly used AFM probe. Using these structural details to infer possible contact angles between the two surfaces at a range of indentation depths, we adjusted the indenter half‐angle value within the Hertzian models to constrain the calculated elastic modulus of PDMS and effectively use it to tune the AFM system.

## 2 MATERIALS AND METHODS

### 2.1 PDMS substrate preparation

PDMS was prepared using SYLGARD® 184 silicone elastomer kit (Dow Corning) containing 10% (w/w) cross‐linking agent The mixture was gently stirred for 1 min with a pipette tip, degassed for ~5 min under vacuum at room temperature and cured at 70°C for 2 h.

### 2.2 Nanoindentation analysis

Freshly prepared and aged samples were analyzed on a TI Premier TriboIndenter® (Hysitron) using a 5 μm *Z*‐axis transducer. The transducer was calibrated at the start of every experiment, following the manufacturers tip to optics calibration routine. The transducer piezo was allowed a rest period prior to PDMS analysis, to minimize drift. Various diamond indenter probes (with differing geometries) and a range of loading forces were used. A Berkovich indenter with a 142.3° included angle and 100 nm radius of curvature was used in deionized water (dH_2_O). Automated and single indents with loading forces of 3–100 μN were taken via load control and displacement control feedback tests. The load function was set to a trapezoid with 5 s load time—50 s hold time—5 s unload time, with a lift height of 10 nm (Figure S1a). A 100 μm radius conospherical probe with a 90° included angle was used in dH_2_O. Single indents were taken at loading forces between 0.8 and 20 μN. The load function was set to a trapezoid with 5 s—2 s—5 s and a lift height of 500 nm–2.0 μm. Tests were performed in air with a 1 μm radius conospherical probe with a 90° included angle. Loading forces of 3–15 μN and a lift height of 180 nm–2 μm were used. The load function was set to a trapezoid with 5 s—50 s—5 s load control. All indentation data were analyzed with TriboScan™ software v. 9.41.0 (Hysitron) and exported into Microsoft Excel (2011) for Mac, v.14.0.0 for further quantification.

### 2.3 Tensile analysis

Five pieces (freshly made and up to 4 weeks old) of PDMS of varying thickness, with a total area between 3 and 12 mm^2^, respectively, were trimmed into rectangles to fit into the specimen grips of a zwickiLine Z0.5 (Zwick Roell). The modulus was determined from a gradient of ~14% on the stress/strain curves, using testXpert® II software (Zwick Roell). The outputted data were exported into Microsoft Excel (2013) v.15.0.5015 for Windows and analyzed further.

### 2.4 AFM multiparametric imaging of PDMS

A NanoWizard® 3 AFM, software v. 5.0.51 was used in Quantitative Imaging (QI™) mode and all images and F‐D curves were analyzed using JPK Data Processing software v.spm‐5.1.13 (JPK Instruments). All experiments were performed in liquid (brain heart infusion broth, 37 g l^−1^) (Fluka) passed through a 0.22 μm filter (Millipore) at 37° C with the same settings. QI™ setpoint was either 1 nN (Figure S2b) or 5 nN. Z‐length was set to 900 nm with an additional 50 nm added before going to the next pixel. Approach and retract times were 40 ms, which equated to 22.5 μm/s. Motion and acceleration times were 1.0 ms and sample rate was 100 kHz (Figure S2a). The resolution was 64 × 64 pixels over a 10 × 10 μm scan region. MLCT silicon nitride cantilevers (Bruker Corporation) with silicon nitride tips were used throughout unless otherwise stated, with a new cantilever used for every experiment. The inverse optical lever sensitivity (InvOLS) (Cleveland et al., 2006) was performed at the start of each experiment in air and liquid on freshly cleaved mica or 1 molar potassium hydroxide‐cleaned glass coverslip. The thermal noise method (Hutter & Bechhoefer, 1993) was used to determine the cantilever spring constant using the calibration routine in the JPK software with corrections applied for the 10° cantilever tilt (Hutter, 2005). The spring constant from the air calibration routine was used with the InvOLS value from the liquid measurements. All *F*–*D* curves were subject to ~200 pN of hydrodynamic drag force. These forces can be problematic for force measurements on soft samples, particularly with oscillating probes. However, there remains predictability with these forces when scanning in the same liquid medium (as in this study), and the drag force dependence on tip speed exhibits linear behavior (Alcaraz et al., 2002; Berthold et al., 2017). The same loading speed was maintained in all our experiments. In principle, we could have subtracted 200 pN from each *F*–*D* curve or performed slower scans. Crucially, however, QI™ mode utilizes a “dynamic baseline adjustment” during imaging, which takes account of hydrodynamic effects, and there is no sinusoidal oscillation of the cantilever, nor feedback loop (Chopinet et al., 2013; JPK Instruments AG, 2011). These combined effects allow for controlled loading on the samples, at all preselected loading rates used in this study, leading to a consistent ~200 pN drag force and accurately defined loads (i.e., 1, 5, 7, and 10 nN) across all *F*–*D* curves (Figure S2–4). Because of the predictability, consistency of experimental conditions and the QI™ corrective factors, we could disregard the hydrodynamic contribution.

### 2.5 AFM indentation method

When formulating the method that led to the adoption of the 85° cone half‐angle settings a range of cantilevers was used for comparative analysis of the PDMS substrate modulus. Standard MLCT cantilevers using the triangular D, E, or F cantilever, or B500_CONTR (nanotools) were used. A range of QI™ setpoints were used (1, 5, 7, or 10 nN) to either ensure that the large spherical indenter reached the same indentation depth of smaller radii tips, or to specifically determine the modulus at increased depths (Figure S4). Freshly made PDMS substrates were scanned at 0.5 nN and immediately after at 1 nN to establish, and subsequently nullify (not shown), if there was a loading force dependence on the reported modulus and the tip geometry at differing indentation depths. Importantly, the tips share the same dimensions across all the cantilevers on the MLCT chip, and so the angle measurements should be consistent from either cantilever. The inbuilt Hertz/Sneddon model in the AFM software allows the user to change the indenter geometry. The Hertz fit for a spherical indenter and the Sneddon fit for a conical indenter were both used. Following the elucidation of the tip angles, a half‐angle of 85° was used for all PDMS modulus data. Indentation of elastic solids has been studied for over a century. Heinrich Hertz first pioneered the contact between elastic bodies (Hertz, 1882) where he approximated shallow indentations for a smooth elastic sphere onto a rigid flat surface, according to the equation

$$F=\frac{4}{\begin{array}{c} 3\\ \; \end{array}}\;\frac{E_{s}}{1-v_{s}^{2}}\;\sqrt{r}\delta^{\frac{3}{2}}$$

where $E_{s}$ is the sample surface modulus, $v_{s}$ is the Poisson's ratio, r is the tip radius of curvature, and δ is the displacement of the indenter. The model was extended to study the contact problem between two linearly isotropic solids (Boussinesq, 1885). Ian Sneddon took the approach by Boussinesq to derive the load–displacement relationship for a rigid conical indenter (Sneddon, 1948) to derive the equation

$$F=\frac{2}{\pi }\frac{E_{s}}{1-v_{s}^{2}}\tan\left(\alpha \right)\delta^{2}$$

where α is the half opening angle of the indenting cone. He later extended his work for other indenter geometries (Sneddon, 1965).

### 2.6 AFM *F*–*D* curve batch processing

All *F*–*D* curves were manually analyzed using JPK Data Processing software v.spm‐5.1.13 (JPK Instruments) using the approach portion of the curves. The inbuilt operators were loaded in a specific, necessary order, and were saved as a user “process” (Figure S3). This process could be easily loaded for all future batch, or individual, curve processing. At least 10 *F*–*D* curves were analyzed for every sample unless otherwise stated. Further adjustment was typically required to better define the contact point, using the “subtract baseline,” “contact point,” and the “correct height for cantilever bending” operators.

### 2.7 Elastic modulus calculations

Often, custom scripts developed with third party software, such as MATLAB, are utilized for the calculation of elastic moduli. These typically employ an equation of contact mechanics, like the Hertz model, and a variety of written code seeks to identify the contact point between probe and sample (Denisin & Pruitt, 2016b; Dhahri et al., 2013). We elected to use the manufacturer software and embedded Hertz models. Each individual *F*–*D* curve was analyzed manually. The position around the zero crossing point was zoomed into and the identification of deflection was considered only where the noise—that deviated above and below the baseline—remained consistently above the baseline. PDMS is relatively stiff (compared to cells) and this made identification of the deflection point easy (Figures S3 and S4). Fortunately, even if this identification under‐ or over‐estimated the exact moment of contact due to the noise, the error is negligible in many AFM applications (Dufrene et al., 2013). The Young's modulus fitting curve within the Hertz/Sneddon operator was applied between this zero‐contact point and −10 nm indentation and the values recorded. Where the fitting curve did not follow the *F*–*D* curve faithfully it may over or underestimate the modulus value. Accordingly, the fitting curve may have been applied to the entire *F*–*D* curve—at indentation depths greater than 10 nm. Where this was required, the reported modulus was checked at multiple points across the *F*–*D* curve for consistency. Where consistency was not evident, these *F*–*D* curves were discarded, and alternative ones used. The values were added into statistical software – both Microsoft Excel (2013) v.15.0.5015 for Windows or Microsoft Excel (2011) for Mac, v.14.0.0. These data were copied into GraphPad Prism version 7.03 for Windows or GraphPad Prism version 8.0.2 for Mac (GraphPad Software Inc., La Jolla, California) for further quantification, statistical analysis, and generation of histograms.

### 2.8 Statistical analysis

Two‐tailed unpaired nonparametric *t*‐test was used, with the Mann–Whitney (Wilcoxon rank sum) test. Where population distributions are presented the averages ± one standard deviation (SD) are shown. All values are reported to 2 significant figures. Significance was considered at an alpha (*α*) level <0.05. For histograms or scatter plots error bars show mean ± SD. Each dot represents an individual *F*–*D* curve.

### 2.9 SEM imaging and micrograph processing

Several uncoated MLCT cantilevers were assayed with SEM (Raith EO). Images were collected at 3.0 kV and aperture size 30.0 μm. All original SEM software‐calculated measurements appear in white. Calibration of the micrograph size was performed by drawing a straight line over a previously SEM‐calibrated marker, such as a scale bar, or cursor region, and using the Analyze—Set Scale option within the Fiji distribution of ImageJ v. 2.0.0‐rc‐65/1.51 s (Schindelin et al., 2012). Various size and angle measurements were added and appear in yellow on the micrographs. The tip apex measurement is in red. Angle measurements were obtained using the angle tool within the Fiji toolbar and using the Analyze—Measure option to find the values. Where sample charging was evident on the detailed AFM tip micrographs, the Process—Find Edges option was used to enable easier visualization of the tip shape. AFM tip images were rotated 180° using the Image—Transform—Rotate option, for easier assessment of indentation angles. Values of resolution, height and width from scaled micrographs were found using the Image—Show Info option. Images were further resized and/or cropped within Microsoft Word where required.

### 2.10 Molecular architecture imaging of PDMS

All high‐resolution imaging was performed on a Dimension FastScan, software v.9.1 (64 bit) (Bruker) in intermittent contact mode using TESPA‐V2 (Bruker) cantilevers in air. Images were captured with a drive amplitude ~47 mV and scan rate was 1.49 Hz. Pixel density was 512 × 512. Z‐range was 0.2–0.5 μm. Calibrations of the phase image size was performed as for SEM, above. To look at the internal structure of PDMS, a ~2 cm^2^ piece was briefly immersed in liquid nitrogen and a pestle was used to crack the PDMS in half. The nonimaged side was carefully sliced to create a level edge, and it was secured to the microscope stage with rubber elastomer (Figure S8). The internally exposed face was scanned in air with a drive amplitude of 160–170 mV and a scan rate of 1.49 Hz. Pixel densities were 512 × 512. *Z*‐range was 0.5 μm. All images were processed with first order (linear) plane fitting to remove any tilt. Streaks or similar artifacts were removed with the relevant manufacturer software routine. Height measurements were taken from streak‐free regions, and prior to removal of any streaks. Where images required more leveling third‐order plane fitting was applied. Where phase images are used the figure legend provides a description. Comparative images were captured on a Dimension 3100 (Veeco) AFM in intermittent contact mode using TESPA‐V2 (Bruker) cantilevers in air, and in QI™ mode on an Ultraspeed (JPK) AFM using Biolever mini (Olympus) probes in deionized water (Figure S10).

### 2.11 PDMS roughness

The top surface and three sub‐surface AFM scans from Figure 4 were quantified using NanoScope Analysis. The arithmetic mean roughness, *R*_a_, the RMS roughness, *R*_q_, and the skewness, *R*_sk_ and kurtosis, *R*_ku_ were calculated from the whole image. All images were processed with third order plane fitting and first order flattened.

## 3 RESULTS AND DISCUSSION

### 3.1 The elastic modulus of PDMS calculated from INI, tensile loading, and AFM

There is a lack of consensus within the literature as to which materials characterization technique best serves a soft sample. Their suitability is hotly debated and further compounded with no clear way of comparing values between different modalities (Kingsley et al., 2019). A common pairing is tension versus compression and compressive moduli are often double those collected from tensile testing. However, compressive tests on PDMS show greater moduli above the 15% strain range which is often at indentation depths up to hundreds of nanometers and beyond (Gaudière et al., 2012; Kingsley et al., 2019; Niu et al., 2018). Strain rates below 15% utilized in this study are within the linear elastic regime for PDMS and this behavior allows the elastic modulus to be calculated via Hooke's law where the tensile stress–strain curve is simply the reverse of the compressive stress–strain curve (Johnston et al., 2014; Niu et al., 2018). When characterized within this regime fiber network models demonstrate identical moduli for both tension and compression (van Dillen et al., 2008).

**FIGURE 1:**
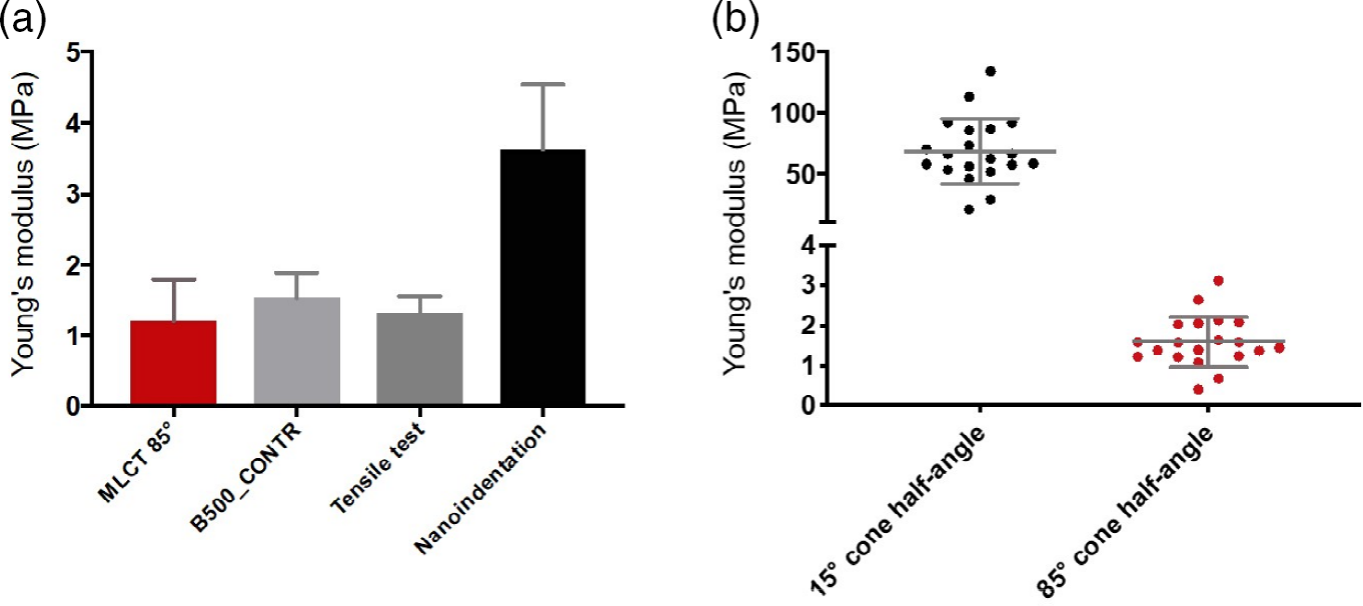
PDMS elastic modulus from a variety of analytical techniques. (a) Elastic modulus comparisons between AFM, tensile loading and INI. (b) Representative scatter plot of the elastic modulus when changing from a nominal conical half‐angle of 15° to a user‐defined half‐angle of 85°

INI relies on the accurate calculation of the indenter tip geometry and elastic moduli are determined by using the Oliver and Pharr model (see Figure S1), which accounts for the changes in contact area at different locations along the unloading portion of an indentation curve (Oliver & Pharr, 1992). The model is based on a conical indenter. INI has reported widely dispersed values of elastic modulus for 10:1 (w/w) elastomer to crosslinker PDMS between ~0.6 and 50 MPa (Charitidis, 2011; Lin et al., 2008; Liu et al., 2009b), with many suggesting an elastic modulus ~3–4 MPa (Deuschle et al., 2008; Shen et al., 2008). In this present work, we tested a variety of PDMS substrates of varying age and thickness (see Section 2), prepared from commercially available SYLGARD‐184. A range of indenters (Berkovich, 1, and 100 μm conospherical), load functions (Figure S1a) and loading forces (2.01–98.2 μN) was utilized, both in a liquid cell, and under ambient conditions (see Section 2). The fluid cell Berkovich analysis using 24 automated indentations in dH_2_O gave us a mean elastic modulus of 3.404 ± 1.383 MPa. Taking two indentations using the 100 μm conospherical probe in dH_2_O gave a mean elastic modulus of 4.635 ± 0.424 MPa. Finally, capturing seven indentations using the 1 μm conospherical probe in air gave a mean elastic modulus of 2.866 ± 0.583 MPa. Combining the data from all the different testing conditions gives a mean elastic modulus of 3.6 ± 0.91 MPa (Figure 1a). The combined mean indentation was 885 ± 740 nm.

INI can be problematic when trying to achieve shallow indentations on a soft substrate, as the loading forces are high and determining the actual point of surface contact is difficult. An insensitivity to the to the initial contact can lead to underestimation of contact depth (Cohen & Kalfon‐cohen, 2013) and it has been shown that PDMS needed to be indented by 2000 nm before 10 μN of force was measured by the system (White et al., 2005). In this present study, we observed similar problems, and it was difficult to determine the exact moment of indentation and thus, an unambiguous measure of elastic modulus or indentation depth. The unloading curve is used to derive elastic moduli, in an attempt to avoid plastic deformations that may be present during loading (Cohen & Kalfon‐cohen, 2013) (Figure S1b). However, the unloading curve of INI and AFM data are subject to adhesion, which is known to lead to overestimation of elastic moduli. Moreover, PDMS does not show plastic deformation, even at indentation depths up to 20 μm (Deuschle et al., 2008).

For tensile loading, five different PDMS substrates were tested. The samples varied in thickness and age—from freshly prepared and up to 4 weeks old (see Methods). The mean sample yielding was at 69% strain, from a maximum applied force at breaking of 3.0 ± 0.56 N. Mean elastic modulus was 1.3 ± 0.23 MPa (Figure 1a) determined from 14% along the stress/strain curve, which is well within the linear elastic region for PDMS (Gaudière et al., 2012; Johnston et al., 2014).

We were interested in the elastic moduli from sub‐100 nm indentations—and particularly 10 nm indentations, as we wished to correlate these data with similarly indented Staphylococcal bacterial cells, which used the PDMS as an immobilizing substrate (not shown). The mechanical properties of the bacterial cell wall, at 25 nm indentations, has shown to be subject to the influence of intracellular turgor pressure (Bailey et al., 2014a) and may not accurately reflect the intrinsic wall material property. We postulated that the bacteria and the PDMS would share similar indentation behaviors, particularly as the molecular architecture of the cell wall (Pasquina‐Lemonche et al., 2020) and the PDMS (this study), share many similarities. Further, we expect that the elastic moduli of both should occupy a similar order of magnitude (Smolyakov et al., 2016b). Thus, accurately defining the tip shape and the elastic modulus of the PDMS should instill some confidence when assessing the elastic modulus of the bacterium. The Bruker MLCT D cantilever was chosen as it is widely used in soft matter studies (Pillet et al., 2016; Smolyakov et al., 2016a). Using the JPK NanoWizard® 3 AFM and software, which utilizes the Hertz/Sneddon model (see Methods) and using the software default conical half‐angle of 15°, gave an elastic modulus of 69 ± 27 MPa from a mean indentation of 10 ± 0.31 nm (Figure 1b). As these moduli were significantly greater than those widely reported for PDMS, and from our previously calculated values, we concluded that the manufacturer designated nominal half‐angle of 17.5° could not hold true at such shallow indentations. Changing between 15° and 17.5° made very minimal change to the calculated moduli (not shown). Adjusting the half‐angle to 85° in the Sneddon model consistently reported the elastic modulus of PDMS at ~1.3 MPa, to match our tensile data (Figure 1a,b). Using the manufacturer defined nominal sphere radius of 20 nm also routinely led to moduli greater than 100 MPa. The radius had to be increased to ~220 nm in the Hertz model, in order to reduce the moduli to ~1.3 MPa (not shown). To address this, a commercially available AFM cantilever (B500_CONTR) with a well‐defined spherical 500 ± 10 nm tip radius was utilized. Multiple PDMS substrates were tested, and 500 nm radius inputted into the model. The mean elastic modulus was 1.5 ± 0.36 MPa (Figure 1a) showing close agreement with the tensile data. Moreover, the large size of this indenter lead to greater adhesion on the F‐D curves, than compared to the MLCT which showed no discernable adhesion at 10 nm indentations (Figure S2b), and greater adhesion often leads to overestimation of elastic moduli. Suriano and colleagues demonstrated differences between contact mechanical models on their analysis of PDMS. Based on a reference modulus of 1.24 ± 0.046 MPa, AFM indentations at ~35 nm reported an elastic modulus of 1.51 ± 0.182 MPa using the DMT model and 1.71 ± 0.069 MPa using the JKR model on the same PDMS samples (Suriano et al., 2014). Following this commonly identified phenomena, and noting the work of Suriano and coworkers, further suggested to us that our PDMS modulus was in the order of ~1.3 MPa. Equally, these results show that the MLCT tip does not behave as a true cone or sphere at sub‐100 nm indentations.

**FIGURE 2:**
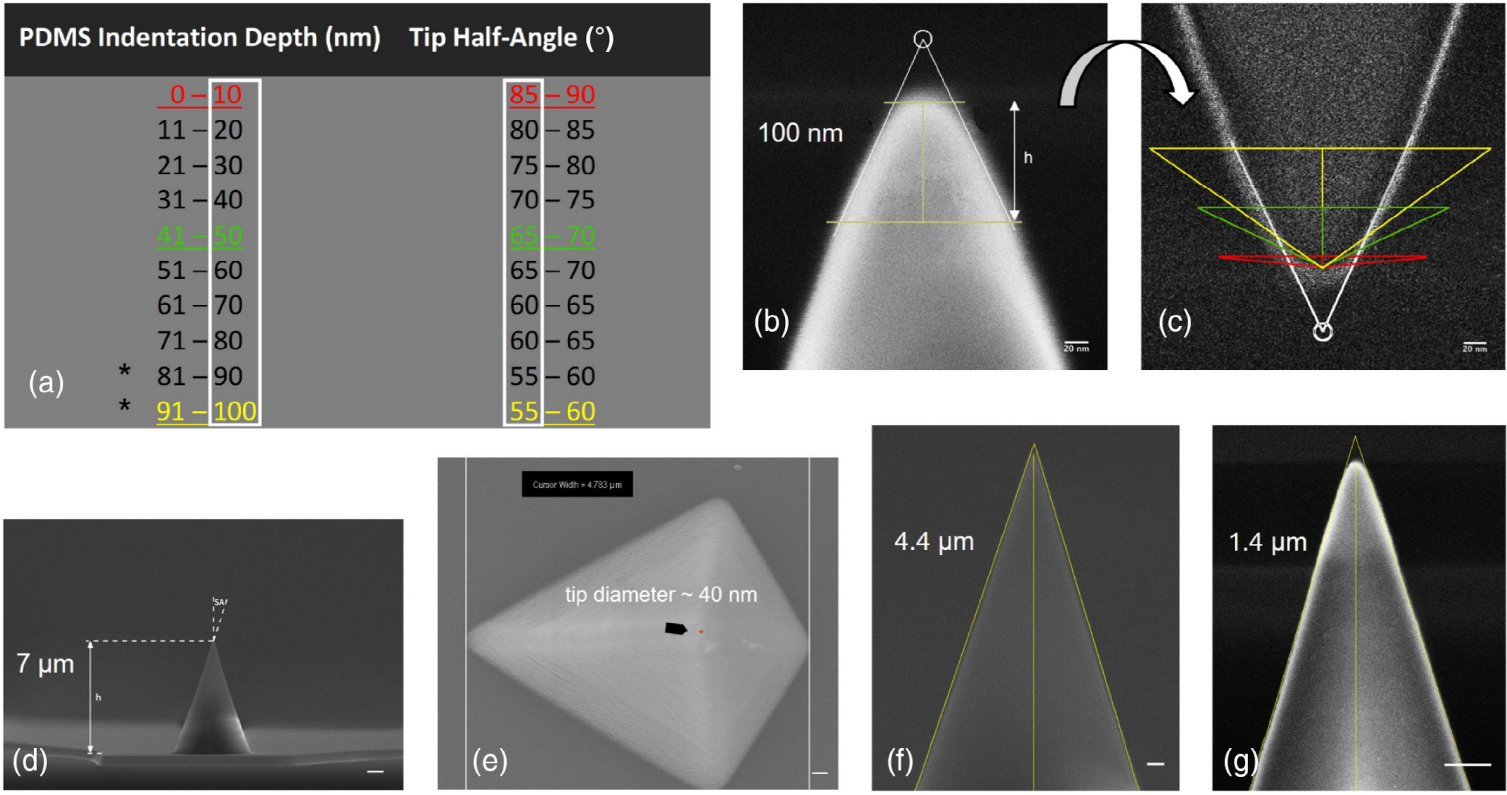
Conical half‐angles as a function of indentation depth and tip height. (a) Sneddon model conical half‐angles required to constrain the PDMS elastic modulus to ~1.3 MPa. The asterisks denote that the angles at these indentation depths were approximated. (b) SEM micrograph of the MLCT tip with emphasis on the top 100 nm. Height and angle lines (white) fitted within the manufacturer software. (c) Rotated copy of (b) using the “find edges” operator within Fiji/ImageJ to identify the charging region around the periphery of the probe. Fitted with conical half‐angles of 85° (red), 65° (green), and 55° (yellow). (d) Full tip height of 7 μm. (e) Top view micrograph depicting the tip diameter (arrowed red circle). (f) Tip height at 4.4 μm from the apex. (g) Tip height at 1.4 μm from the tip apex. Yellow lines drawn for height measurement in b, and height and angle measurements in f and g. scale bar in (b,c), 20 nm; in (d) 1 μm; and (e–g) 200 nm

A range of loading forces and stiffer MLCT cantilevers was utilized to enable indentations up to 79 nm. We postulated that the modulus would remain the same up to 100 nm indentations. Conical half‐angles were adjusted within the Sneddon model, to constrain the elastic modulus at ~1.3 MPa. A somewhat linear trend of increasing depth and concomitant decreasing half‐angles was observed (Figure 2a). To reduce ambiguity, and to simplify the data analysis, indentations were ordered into 10 nm groupings and half‐angle values into 5° groupings. Thus, 10 nm indentations were fitted with an 85° half‐angle. As 79 nm was the maximum indentation that we could achieve, we approximated the 80–100 nm indentation depth half‐angles at 55°–60° by following the previous indentation and angle trends (Figure 2a). The loading rate of 22.5 μm/s (see Section 2) was maintained with all probes and loading forces used. Viscoelastic materials, like PDMS, exhibit stress relaxation behavior under compressive loading as a function of time, although the effect is limited and declines rapidly, reaching a steady state (Zhang et al., 2022). When loading rates are low (e.g., 2 μm/s) the molecules are able to move and recover to their original conformation, whereas for faster loading rates (e.g., >500 μm/s) the molecules cannot move fast enough to follow the induced deformation, and subsequently the material behaves more like a stiff material rather than an elastic one—leading to increased elastic moduli (Kim et al., 2008). It has been shown that PDMS exhibited no time‐dependent effects on loading curves when indented at speeds between 0.2 and 200 mm/min (i.e., 3–3300 μm/s) (Lim & Chaudhri, 2004). Our loading rate, whilst not considered fast for the QI™ method may be considered quick for different AFM systems, and researchers should examine their *F*–*D* curves for suitability of fit—particularly if any hydrodynamic drag is unaccounted for. If adhesion was unavoidable, a modified version of the Hertz model (Carl & Schillers, 2008) or a different model that takes account of adhesion, such as the JKR model (Lin & Kim, 2012) may be a better solution.

### 3.2 SEM reveals changes in geometry toward the tip apex

Because the elastic moduli calculated from the spherical B500_CONTR probe and tensile loading were in close agreement we sought to identify the conical half‐angle and radius of the MLCT tip in greater detail, to determine if our approximated conical half‐angles were realistic. Typically, samples are coated with a metal to reduce any charging effects and to provide good contrast. However, the coating is often 10–20 nm in thickness, and often not of a uniform distribution (Figure S5). Given that the tip diameter is only around 40 nm (Figure 2b,c,e) this could be problematic. As we were interested in the terminal 10 nm from the tip apex, we used uncoated tips and obtained high‐resolution micrographs with only moderate signs of charging (Figure 2b–g). The manufacturer assigned a tip height between 2.5 and 8.0 μm and a side angle of 17.5° ± 2.5°. We measured a tip height of ~7 μm, and the side angle was approximated as 16.3°, in close agreement with the manufacturer (Figure 2d). The angle was found to change very little when increasing the magnification to show ~4.4 μm (Figure 2f) and ~1.4 μm from the tip apex (Figure 2g) with half‐angle measurements of 17.8° and 17.2°, respectively. When increasing the magnification to show the top 250 nm of the tip the angle became much shallower, and the tip apex appeared broader and flatter, with a spherical end cap. Using the SEM software, an angle of 50° was measured at around 100 nm from the tip apex (Figure 2b,c). Figure 2c shows a micrograph edited with the “Find Edges” operator within Fiji/ImageJ to identify the charging region around the periphery of the probe (see Section 2). Rotating it 180° enables easier visualization of its capacity as a vertical indenter. The approximated conical half‐angle values of 85°, 65°, and 55° respectively (from Figure 2a), were fitted exactly from the tip apex to represent the contact geometry between the probe and PDMS that would be required to constrain the elastic modulus at ~1.3 MPa, when indenting at 10, 50, and 100 nm, respectively.

Our method suggests that at 10 nm indentations the full angle had to be adjusted to nearly 180° representing an almost flat punch contact area between the probe and the sample. As the probe is indented further, the angle becomes steeper as more material begins to contact the sides of the probe, which seems logical. Materials either sink‐in or pile‐up when indented. A soft metal, such as aluminum has been shown to pile‐up during indentation (Van Vliet et al., 2004). Conversely, Deuschle and coworkers extensively studied 10:1 (w/w) PDMS using a combination of INI, SEM, AFM, and optical microscopy. They used a cube corner probe and found that even at 15 μm indentations a clear sink‐in effect was observed, and that the shape of the impression, rather than being pyramidal, was more conical. All of their indentations recovered fully, confirming true rubber‐like properties of PDMS (Deuschle et al., 2008).

### 3.3 Characterizing the molecular architecture and sink‐in of PDMS with AFM and optical microscopy

**FIGURE 3:**
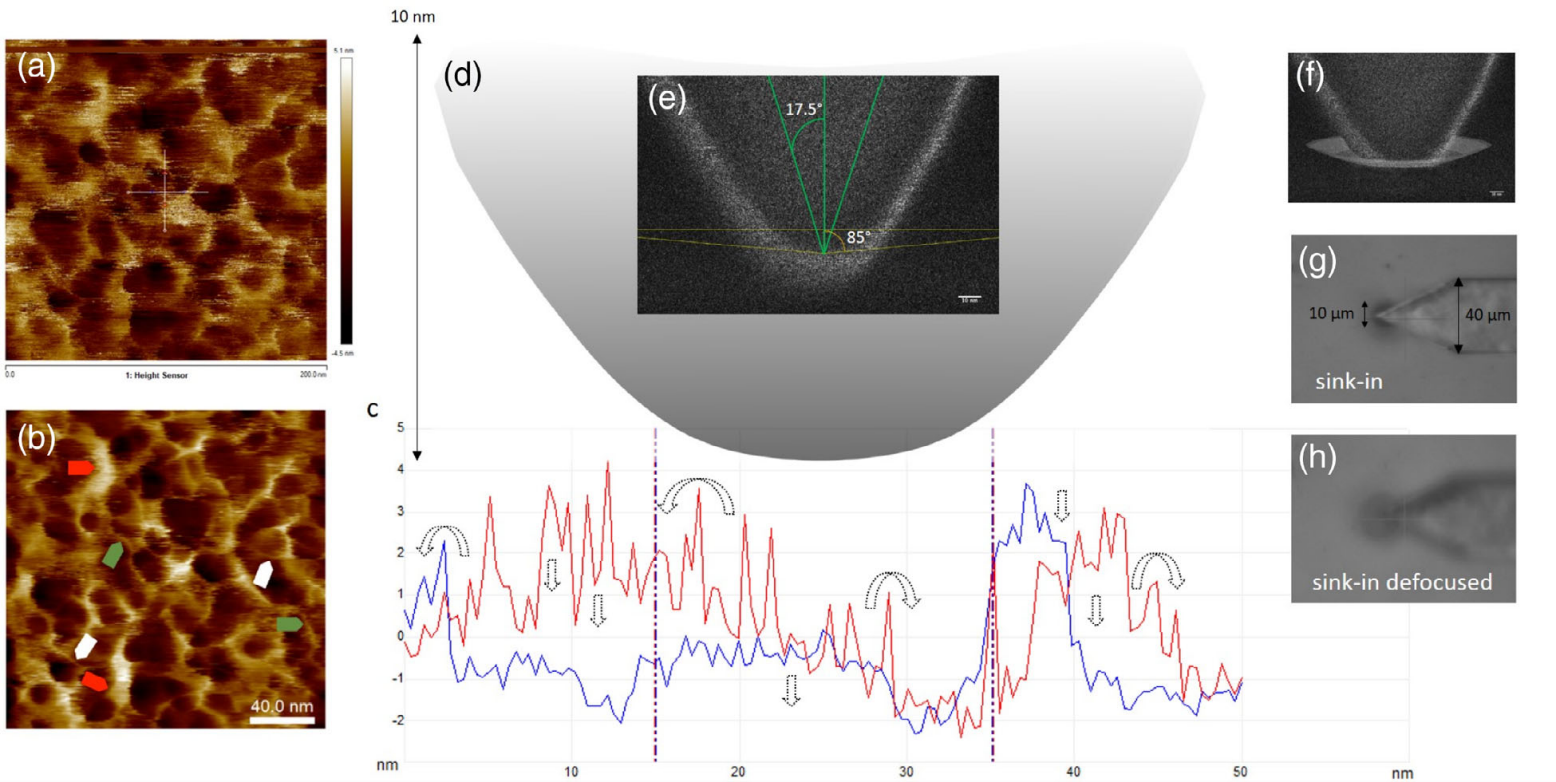
The molecular architecture of PDMS and its interaction with the tip apex. (a) 200 × 200 nm AFM topograph with 50 nm line profiles drawn in *x* (blue) and *y* (red), as shown in (c). (b) Corresponding scaled phase image with polymer strand widths of approximately 1 and 2 nm, and bundle width measurements of 10 nm (white, green, and red arrows respectively). Phase angle (dark to light)‐4.9° to 9.5°. (c) Section profiles from (a). Arrows denote possible deformation of asperities and bundles. (d) MATLAB modeled tip (from SEM micrograph measurements) with a 20 nm diameter at the apex, increasing to 40 nm diameter 10 nm above the apex. (e) SEM micrograph of the leading 90 nm up to the tip apex. Yellow lines show an 85° half‐angle fitted at a measured 10 nm from the leading edge of the tip. Green overlaid lines show the assumed nominal 17.5° half‐angle. (f) Modified copy of (e) with cartoon depicting the possible sink‐in deformation of PDMS required to account for an 85° half‐angle contact geometry at 10 nm indentation. Images e and f were rotated 180° for easier visualization (scale bars, 10 nm). (g) Optical micrograph showing sink‐in deformation caused by an AFM probe. (h) Defocused image to show the diameter of sink‐in curvature in better detail

A high resolution AFM topograph of 10:1 (w/w) PDMS shows the surface to be highly porous (Figure 3a) and the phase channel image (Figure 3b) highlights structural details with greater clarity. Measurements on the scaled phase image (see Methods) show strands of approximately 1 nm (white arrows) and 2 nm widths (green arrows) with dense bundles of ~10 nm widths (red arrows), which correlate with the expected structures (Granick et al., 2003; Yamada, 2003). A 50 × 50 nm profile in *x* and *y* was produced to examine the topography and surface asperities (Figure 3c) and depictions of how these might be deformed or flattened under loading—to account for an almost flat punch contact geometry—are represented via dotted arrows (Figure 3c). The tip apex (Figure 3d) was modeled from measurements taken from high magnification SEM micrographs (Figure 3e,f). The approximated half‐angle of 85° was fitted at 10 nm from the tip apex and a 17.5° half angle is overlaid, demonstrating the inaccuracy of that measurement at this length scale. A cartoon representation of how the PDMS would need to deform to allow for such a shallow contact angle (Figure 3f) is strikingly similar to the deformation of PDMS assessed by Deuschle and coworkers, whose data showed that the contour of the contact area was bowed, and larger than the true contact area (Deuschle et al., 2008). They performed large indentations between 5 and 20 μm and our approximations suggest a similar relationship at the nanoscale. To further corroborate the microscale deformation of PDMS we observed a large sink‐in depression when indenting with a stiff tapping mode probe (Figure 3g,h) which grew considerably larger with increased loading (Figure S6b,c). It should be noted that indenting with the softer MLCT cantilevers did not produce a visible sink in, compared to those observed in Figure 3g,h, using our top mounted optics. It is expected that any sink‐in would naturally start small at the nanoscale, as depicted in the cartoon in Figure 3f, and grow increasingly larger under a greater load. Thus, this nanoscale sink‐in would be obscured by the greater size of the cantilever when viewed from above. Deuschle and colleagues also utilized finite element analysis to infer possible contact area between their sample and indenter, which showed good agreement (albeit slightly smaller), with their experimental contact mechanics model (Deuschle et al., 2008). In this regard, the use of finite element modeling could be explored in future work as it may help to address some of the limitations of this study and potentially offer new insights into the deformation of soft materials at shallow indentations.

### 3.4 Internal architecture of PDMS

**FIGURE 4:**
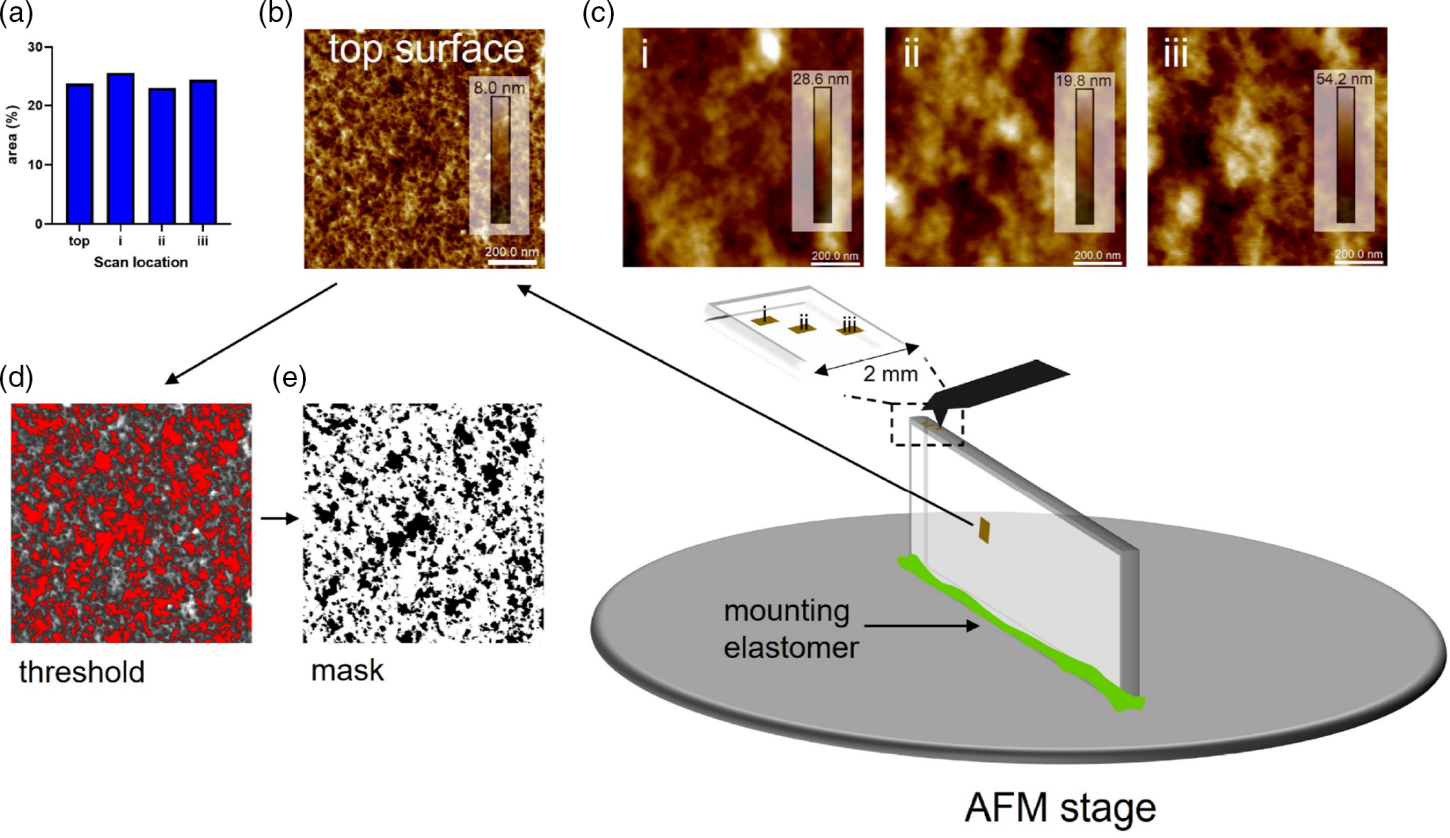
Internal structure of PDMS. The sample was snap‐frozen and broken open exposing the internal structure. (a) area of porosity (%) comparison between the surface and sub‐surface. (b) AFM topograph of the surface. (c) Sub‐surface structure at (i) nm, (ii) μm, and (iii) mm depths from the surface. (d) Representative Fiji/ImageJ threshold and (e) mask created for porosity calculations. All images are 1 × 1 μm scan size. Image pixel density, 512 × 512. *Z* scale (dark to light) inset in (b, c)

The molecular organization of the surface (Figure 4b) was compared to the molecular organization deeper within the 10:1 (w/w) PDMS at nm (Figure 4c i), μm (Figure 4c ii) and mm (Figure 4c iii) depths by moving the probe along the exposed internal surface, which was created by snap‐freezing in liquid nitrogen and cracking open (see Section 2 and Figure S8). Initial scans were performed on a blade‐sliced sample (Figure S7), but the steep cutaway led to some difficulties during scanning. The PDMS was secured to the AFM stage with the internal surface facing upwards towards the probe. Optically, there were numerous, seemingly ordered, and slightly concentric lines evenly dispersed throughout the interior (Figure S7 and S8). Areas between these lines were scanned with the AFM. The molecular architecture at all depths appeared to be similar to the surface, but with a greater number of dense bundles (Figures 4c, S7, and S9). The internal structure did not look as porous as the surface and there were deeper regions ranging from ~20 nm (Figure 4c ii) to ~54 nm (Figure 4c iii) than compared to the maximum depth of 8 nm at the surface (Figure 4b). The porosity was quantified using the Analyze Particles command within Fiji/ImageJ (see Section 2 and Figure S9) by first creating a grayscale image and adjusting the threshold (Figure 4d), followed by creating a mask (Figure 4e and S9), which then allows for an area percentage to be assessed. All images shared a similar mean percentage of 24.2 ± 1.06 (Figure 4a). Although the internal structure does not initially appear to be as porous as the surface, the increased depth likely added to the area percentage calculations. Structurally, the apparent reduction in porosity could be due to the image contrast in the AFM scans. We binary filtered the internal structure images, and these more closely resembled the top surface (Figure S9).

### 3.5 Roughness measurements at the surface and subsurface

To further understand how the PDMS and the AFM probe interact at sub‐100 nm indentations we analyzed the roughness at the surface and the subsurface from the whole image (Table 1). *R*_a_ and *R*_q_ both represent surface roughness and refer to variations in the height of a surface relative to a plane of reference. They are standard measures used in engineering and tribology. *R*_a_ is calculated as the arithmetic average of the peaks and valleys, and *R*_q_ is the root mean square (RMS) of the same measurements. A single large peak or valley would raise the *R*_q_ value more than the *R*_a_. The maximum roughness *R*_max_ is a measure of the largest single depth established from the sample. The skewness and the kurtosis are included to infer information about the Gaussian distributions. Both values are dimensionless. Positive values, away from zero, show a distribution with tails to the right, and negative values show a distribution to the left. A kurtosis of 3 represents normal peakedness of the distribution (Bhushan, 2001). Thus, for example, the top surface has a Gaussian distribution with almost zero skewness and a kurtosis close to 3. This represents an equal number of peaks and valleys. Values >3 suggest more peaks and <3 more valleys.

**TABLE 1:** Surface and sub‐surface roughness measurements of PDMS calculated from 1 × 1 μm AFM topographs

	*R*_a_ (nm)	*R*_q_ (nm)	*R*_max_ (nm)	*R*_sk_	*R*_ku_
Top surface (Figure 4b)	1.1	0.9	12.0	0.1	3.3
Sub‐nm (Figure 4c i)^a^	2.8	3.8	33.0	0.7	4.8
Sub‐μm (Figure 4c ii)	2.2	2.8	22.4	−0.2	3.2
Sub‐mm (Figure 4c iii)	6.8	8.3	48.4	0.2	2.6

^a^ This sub‐surface scan only, being within a few hundred nanometers of the top surface, is likely to influence sub‐100 nm indentations. *R*_a_ is the arithmetic average, *R*_q_ the root‐mean‐square, *R*_max_ is the maximum roughness. *R*_sk_ is skewness and *R*_ku_ is kurtosis.

It can be seen from Figure 4c and the tabulated data in Table 1, that the subsurface is rougher and denser than the top surface of PDMS, with a mean roughness of 3.9 ± 2.5 nm. However, at sub‐100 nm indentations, and most certainly at 10 nm indentations, it will be the surface architecture that bears the greatest impact on the contact geometry between an indenting probe, and thus, the reported moduli from modifications to a contact mechanics model, such as the Hertz/Sneddon model utilized in this work. The small, but numerous asperities that we postulate would be pushed aside, coupled with the bulk sink‐in effect of PDMS, may explain the almost flat punch‐like contact geometry on a seemingly spherical tip apex, as less material would be in contact with the probe at very small indentations.

## 4 CONCLUSIONS

This study sought to better understand the contact geometry relationship between an indenting probe and PDMS, and how the molecular properties of the polymer may lead to contact angles that differ to the expected values. We compared the molecular architecture of PDMS at the surface and within the bulk material. Our data suggest that the porosity is similar between the surface and the interior, but that the PDMS may be slightly denser within the bulk material, with more apparent bundles. Using the classical Hertzian contact mechanics model, we identified that adjusting the half‐angle geometry of the indenter tip has a marked influence on the reported moduli of the sample under investigation. Using high magnification SEM of an AFM probe we showed that the shape changes markedly toward the tip apex and the progressively steeper narrowing away from the apex show a similar trend to our approximated angles. Ultimately, we used the conical half‐angle as an adjustable parameter and fixed it at a range of indentation depths (from 0 to 100 nm) to constrain the elastic modulus of PDMS at ~1.3 MPa, which we calculated from tensile loading and from a spherical probe with a well‐defined radius. To this end, the PDMS was used as a calibrant to infer the AFM tip geometry. With a reasonable knowledge of the probe geometry, and the sample properties, this method may allow the investigator a simple method to improve the mechanical quantification of a variety of soft materials, and minimize the often widely dispersed data reported in the literature.

## CONFLICT OF INTEREST

There are no conflicts to declare.

## Supporting information


**Supplementary Figure S1.** INI load function and indentation curve on PDMS. (a) A representative trapezoidal load function at 5 μN with 5 s load – 50 s hold – 5 s unload. (b) A representative loading/unloading curve with a 1000 nm lift height and maximum indentation of 870 nm. The reduced elastic modulus is obtained by fitting a tangent to the steepest portion of the unloading curve. This is applied by the software. A representative depiction is manually drawn here in red.
**Supplementary Figure S2.** QI™ loading time and F‐D curve on PDMS. (a) Representative loading function of a QI™ indentation at 1 nN loading force. All elastic moduli from 10 nm indentations were derived under the same loading conditions. A loading force (setpoint) of 1 or 5 nN and an approach and retract speed of 22.5 μm/s equated to an approach and retract time of 40 ms with the surface contact taking ~2.5 ms. (b) Representative F‐D curve on PDMS at 1 nN loading force. There was a typical separation of ~200 pN between the approach and retract portions of the F‐D curves which were due to hydrodynamic drag but were discounted as these were a constant throughout all experiments.
**Supplementary Figure S3.** Representative raw data F‐D curves depict the process of applying the AFM software operators. These allow for accurate reporting of sample height, indentation depth and elastic modulus. (a) Annotated curve depicting the approach (light blue curve) when the probe is above the surface; deflection (combined deflection of the probe and indentation of the sample once contact is made, and until the loading threshold is met); retract (dark blue curve) when the probe lifts from the surface and moves away. (b) The approach and retract curves are separated from each other due to hydrodynamic viscous drag. The raw curve is adjusted to bring the (b) baseline offset (linear portion of the approach curve) towards zero. (c) The contact location between tip and sample is approximated. (d) The cantilever deflection is accurately determined. The Hertzian fitting consistently showed that selecting the (e) conical fit produced more faithful fitting of the F‐D curve than when compared to (f) a spherical fit. All the other indenter geometries available in the software were explored—sphere, paraboloid, triangular pyramid, quadratic pyramid and flat cylinder. Editing the radii or half‐angle in these parameters did not provide the expected modulus values of ~ 1.3 MPa and/or apply a sufficient fit to a F‐D curve.
**Supplementary Figure S4.** Representative raw data analysis of PDMS moduli at sub‐100 nm indentations. F‐D curve conical angle fittings on PDMS. Loading forces of 1, 5, 7 and 10 nN were used to indent PDMS to progressively increasing depths and the conical half‐angles were adjusted to maintain an elastic modulus ~1.3 MPa (circled in red in each image). Annotated curves (white arrows) depicting the region of the F‐D curve used for (a) calculation of elastic modulus (b) shows the adhesion energy which is disregarded in the Hertzian models and known to lead to slight overestimation of elastic modulus. Indentation depths were (a) 8 nm (b) 36 nm (c) 48 nm (d) 58 nm (e) 67 nm. To check for any ambiguity with actual indentation contact points the same F‐D curves were routinely checked at different contact points to adjust the slope fitting (visible as the light gray background area which contrasts against the unfitted dark gray region in all raw F‐D plots); represented in (c, d) (dashed yellow arrows) and the conical half‐angle was adjusted to maintain a modulus ~1.3 MPa as a result of the concomitant change in indentation depth. Large adhesion forces were unavoidable with increased loading and indentation and represent a limitation in our model. Black arrows in (b,d,e) show the hydrodynamic drag force remains constant at ~200 pN at all preselected loading forces.
**Supplementary Figure S5.** Sputter‐coated tips show a rough morphology. Initial SEM was performed with a ~ 10 nm layer of gold sputter‐coated onto the AFM tip, but the tip was seen to adopt a rough morphology with rounded clumps of coating visible at all length scales. The parallel lines in Figure a denote the top 10 nm from the tip apex and were applied within the manufacturer software.
**Supplementary Figure S6.** Optical images of AFM probe‐induced sink‐in on PDMS. Top‐down optical micrographs of a tapping probe interacting with 10:1 (w/w) PDMS. The top row shows images focused on the probe and the bottom row shows defocused images centered at the sink‐in to better display the indentation area. Dashed blue lines encircle the deformed regions (a) Above the PDMS surface with no contact. (b) Moderate load and deflection of the probe and the concomitant sink‐in effect on the PDMS. (c) High load and deflection with greater sink‐in deformation continuing along the length of the probe.
**Supplementary Figure S7.** Analysis of the molecular architecture of PDMS at the surface and sub‐surface. (a) A TESPA‐V2 cantilever is positioned near a blade‐sliced surface and sub‐surface interface prior to AFM image capture of 10:1 (w/w) PDMS. Scale bar is 100 nm. Inset (white dotted box) shows a magnified region. (b) Sliced PDMS AFM phase image of surface and sub‐surface. The thick line (arrowed) represents the interface, and the AFM was not able to sufficiently capture image data, due to the steep angle. The vertical white line represents a line profile fitted to the AFM height image of the same data. (c) The line profile data report a sub‐surface depth ~ 100 nm. Image was 3rd order flattened. Phase angle (dark to light) = − 28.4° to 5.7°. Image size = 1 × 1 μm.
**Supplementary Figure S8.** Snap‐freezing method for obtaining μm and mm sub‐surface AFM images. Exposed internal structure of PDMS prior to AFM analysis. PDMS has been briefly immersed for 20 s in liquid nitrogen and cracked to expose a smooth internal surface. (a) The PDMS was secured to the microscope stage (white arrow) and the exposed internal surface was oriented upwards for AFM analysis. The light from the illuminator has created a green reflection from the mounting elastomer. The cantilever is mounted in the *Z* scanner connector (red arrow). (b) An optical micrograph shows a TESPA‐V2 cantilever above the internal surface. Multiple concentric lines appear to be evenly distributed throughout. Scale bar = 100 nm.
**Supplementary Figure S9.** Threshold mask and edited AFM images of the PDMS sub‐surface. (a‐c) Fiji/ImageJ Analyze particles—mask outputs used to estimate the porosity of the sub‐surface scans i, ii, and iii respectively (from Figure 3c). (d‐e) To reveal more structural details the same AFM images used for the above processing have been binary filtered within Fiji/ImageJ to remove pixels from the edges of black objects with 8, 10 and 8 pixel sizes respectively. *Z* scale = (d) 32.6 nm (e) 20.5 nm (f) 57.7 nm. Phase angle (dark to light) = (d) 26.4° to 34.0° (e) ‐21.4° to 31.4° (f) 29.7° to 41.7°. Image pixel density = 512 × 512. All images are 1 × 1 μm scan size.
**Supplementary Figure S10.** PDMS architecture from alternative AFMs and conditions. Comparisons against the molecular architecture of PDMS observed using the Bruker FastScan in air (Figures 3 and 4 and S7b). (a) 1 × 1 μm topograph using a Dimension 3100 (Veeco) AFM in intermittent contact mode, with TESPA‐V2 (Bruker) cantilevers in air. (b,c) 1 × 1 μm and 0.5 × 0.5 μm topographs, respectively, in deionized water using a Biolever mini (Olympus) probe in QI™ mode on an Ultraspeed AFM (JPK).

## Data Availability

The data that support the findings of this study are available from the corresponding author upon reasonable request.
