# Supplementary material for: Toward a better modulus at shallow indentations—Enhanced tip and sample characterization for quantitative atomic force microscopy
Source: Microsc Res Tech. 2022 Nov 18;86(1):84–96. doi: 10.1002/jemt.24261 (PMC10099859; doi:10.1002/jemt.24261)
Supplement: Supplementary file 1 — Supplementary Figure S1. INI load function and indentation curve on PDMS. (a) A representative trapezoidal load function at 5 μN with 5 s load – 50 s hold – 5 s unload. (b) A representative loading/unloading curve with a 1000 nm lift height and maximum indentation of 870 nm. The reduced elastic modulus is obtained by fitting a tangent to the steepest portion of the unloading curve. This is applied by the software. A representative depiction is manually drawn here in red. Supplementary Figure S2. QI™ loading time and F‐D curve on PDMS. (a) Representative loading function of a QI™ indentation at 1 nN loading force. All elastic moduli from 10 nm indentations were derived under the same loading conditions. A loading force (setpoint) of 1 or 5 nN and an approach and retract speed of 22.5 μm/s equated to an approach and retract time of 40 ms with the surface contact taking ~2.5 ms. (b) Representative F‐D curve on PDMS at 1 nN loading force. There was a typical separation of ~200 pN between the approach and retract portions of the F‐D curves which were due to hydrodynamic drag but were discounted as these were a constant throughout all experiments. Supplementary Figure S3. Representative raw data F‐D curves depict the process of applying the AFM software operators. These allow for accurate reporting of sample height, indentation depth and elastic modulus. (a) Annotated curve depicting the approach (light blue curve) when the probe is above the surface; deflection (combined deflection of the probe and indentation of the sample once contact is made, and until the loading threshold is met); retract (dark blue curve) when the probe lifts from the surface and moves away. (b) The approach and retract curves are separated from each other due to hydrodynamic viscous drag. The raw curve is adjusted to bring the (b) baseline offset (linear portion of the approach curve) towards zero. (c) The contact location between tip and sample is approximated. (d) The cantilever deflection is ac [file JEMT-86-84-s001.docx]

**Supplementary Information**

Towards a Better Modulus at Shallow Indentations – Enhanced Tip and Sample Characterization for Quantitative Atomic Force Microscopy

David S. Owen

Department of Physics and Astronomy, University of Sheffield, Sheffield, South Yorkshire, S3 7RH, U.K. E-mail: david.owen@sheffield.ac.uk

**Oliver and Pharr contact mechanics model.** To obtain values of elastic modulus from INI the authors assume that the indenter geometry can be described by an area function $F(h)$ which relates the cross-sectional area of the indenter to the distance from its tip. The projected contact area, $A$ at peak load can be computed from the relation $A=F\left( h_{c} \right)$ where $F$ is determined experimentally prior to analysis and $h_{c}$ is the contact depth. To correct for any effects of non-rigid indenters on the load-displacement behavior a reduced elastic modulus, $E_{r}$ can be defined according to the equation

$\frac{1}{E_{r}}= \frac{\left( 1-v_{i}^{2} \right)}{E_{i}} +\frac{\left( 1-v_{s}^{2} \right)}{E_{s}}$

where $v$ is the Poisson’s ratio and $i$ and $s$ denote the indenter and sample respectively.

As the elastic modulus of the PDMS is orders of magnitude lower than that of the indenter, the indenter term can be neglected (Ebenstein & Pruitt, 2004). The reduced elastic modulus could be solved according to the equation

$E_{r}= \frac{\sqrt{\pi}S}{2\sqrt{A}}$

where the stiffness, $S$ is experimentally measured from the upper portion of the unloading curve according to the derivatives of the indenter load with respect to the indenter displacement $\left( dF/dh \right)$. This holds for any indenter that can be described as a body of revolution, and thus, is not limited to a specific geometry (Oliver & Pharr, 1992). In the formulation of their contact model Oliver and Pharr made the assumption that the elastic modulus was independent of indentation depth. Further, they defined a geometric constant for various indenter geometries. A flat punch was defined as 1, a paraboloid as 0.75 and a conical indenter as 0.72. They found that the paraboloid better described the unloading behavior but note that even if a conical geometry had been chosen, the only difference in their analysis would have been to use the slightly smaller constant of 0.72 (Oliver & Pharr, 1992).

| a | b |
| --- | --- |
|  |  |
| Supplementary Figure S1 INI load function and indentation curve on PDMS. (a) A representative trapezoidal load function at 5 µN with 5 s load – 50 s hold – 5 s unload. (b) A representative loading/unloading curve with a 1000 nm lift height and maximum indentation of 870 nm. The reduced elastic modulus is obtained by fitting a tangent to the steepest portion of the unloading curve. This is applied by the software. A representative depiction is manually drawn here in red. | |

| a | b |
| --- | --- |
|  |  |
| Supplementary Figure S2 QI™ loading time and F-D curve on PDMS. (a) Representative loading function of a QI™ indentation at 1 nN loading force. All elastic moduli from 10 nm indentations were derived under the same loading conditions. A loading force (setpoint) of 1 or 5 nN and an approach and retract speed of 22.5 µm/s equated to an approach and retract time of 40 ms with the surface contact taking ~ 2.5 ms. (b) Representative F-D curve on PDMS at 1 nN loading force. There was a typical separation of ~ 200 pN between the approach and retract portions of the F-D curves which were due to hydrodynamic drag but were discounted as these were a constant throughout all experiments. | |

| a | b 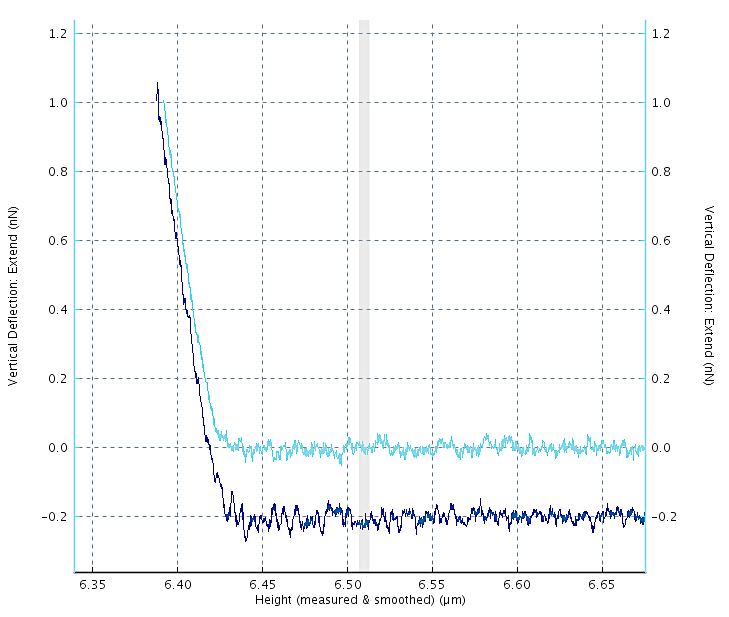 hydrodynamic drag |  |
| --- | --- | --- |
| 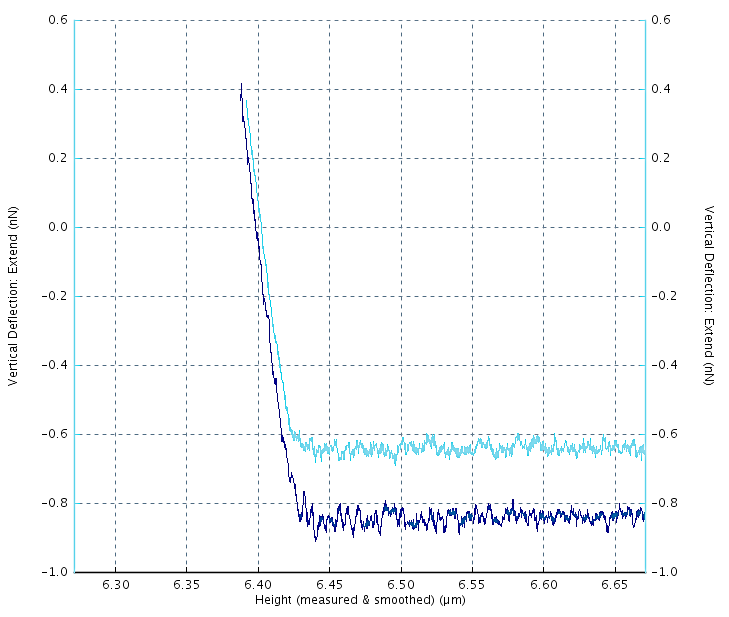  deflection  approach  retract  retract | hydrodynamic drag |  |
| c | d |  |
| 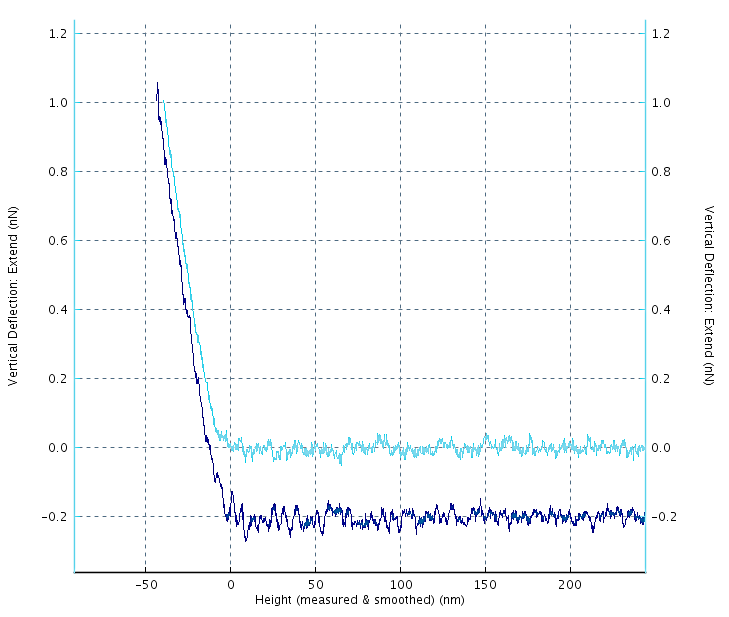 | 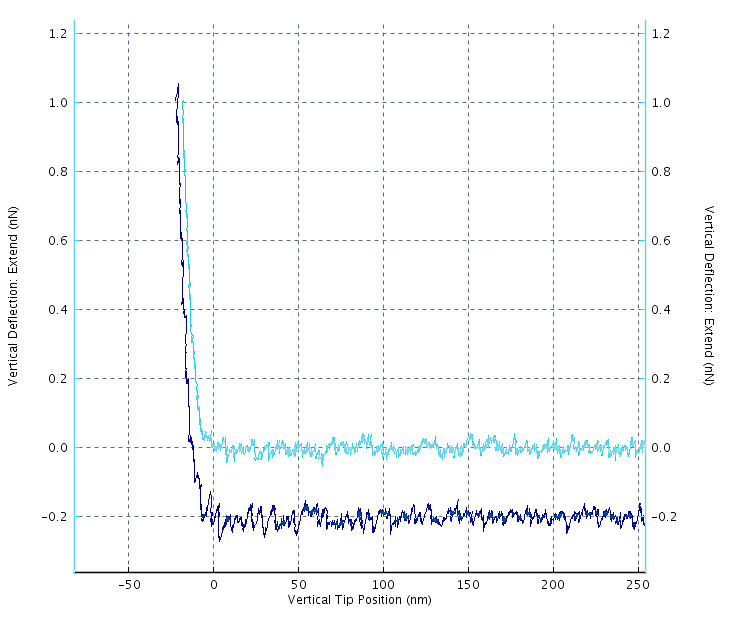 |  |
| e 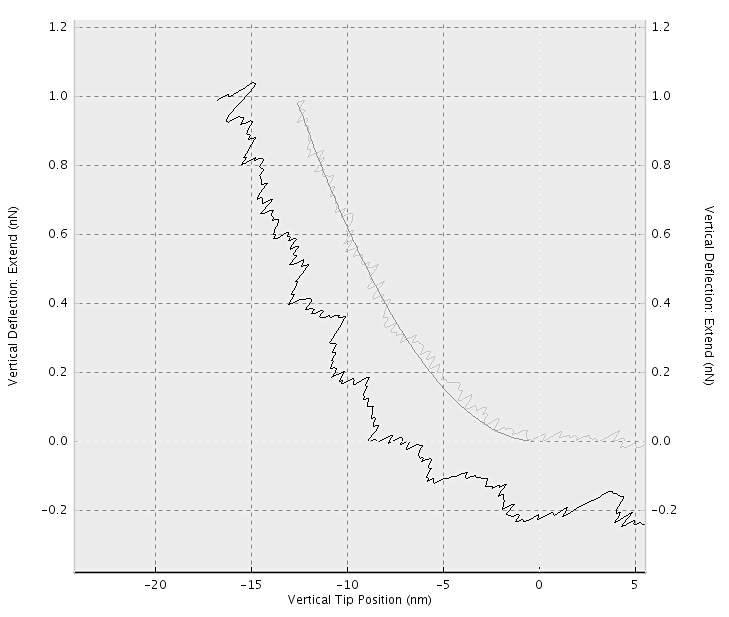 elastic modulus | f |  |
|  | 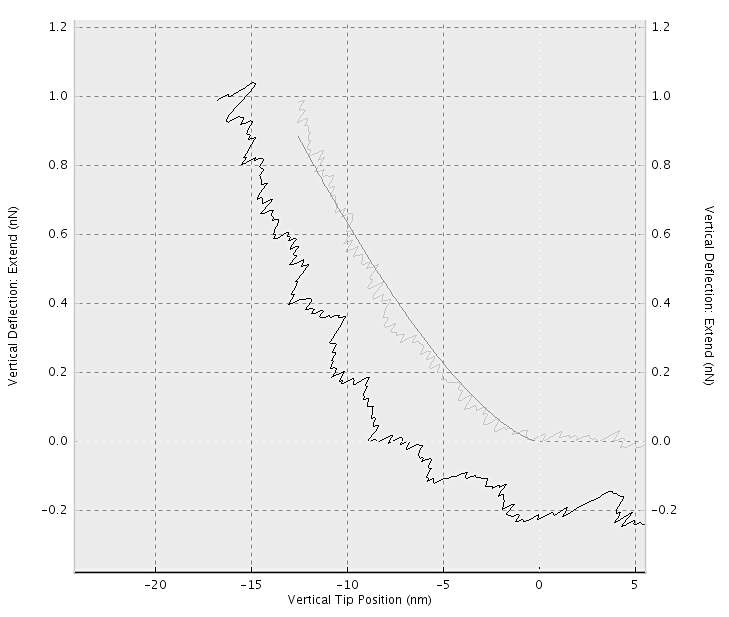  indentation depth |  |
| Supplementary Figure S3 Representative raw data F-D curves depict the process of applying the AFM software operators. These allow for accurate reporting of sample height, indentation depth and elastic modulus. (a) Annotated curve depicting the approach (light blue curve) when the probe is above the surface; deflection (combined deflection of the probe and indentation of the sample once contact is made, and until the loading threshold is met); retract (dark blue curve) when the probe lifts from the surface and moves away. (b) The approach and retract curves are separated from each other due to hydrodynamic viscous drag. The raw curve is adjusted to bring the (b) baseline offset (linear portion of the approach curve) towards zero. (c) The contact location between tip and sample is approximated. (d) The cantilever deflection is accurately determined. The Hertzian fitting consistently showed that selecting the (e) conical fit produced more faithful fitting of the F-D curve than when compared to (f) a spherical fit. All the other indenter geometries available in the software were explored - sphere, paraboloid, triangular pyramid, quadratic pyramid and flat cylinder. Editing the radii or half-angle in these parameters did not provide the expected modulus values of ~ 1.3 MPa and/or apply a sufficient fit to a F-D curve. | |  |
|  |  | |
| 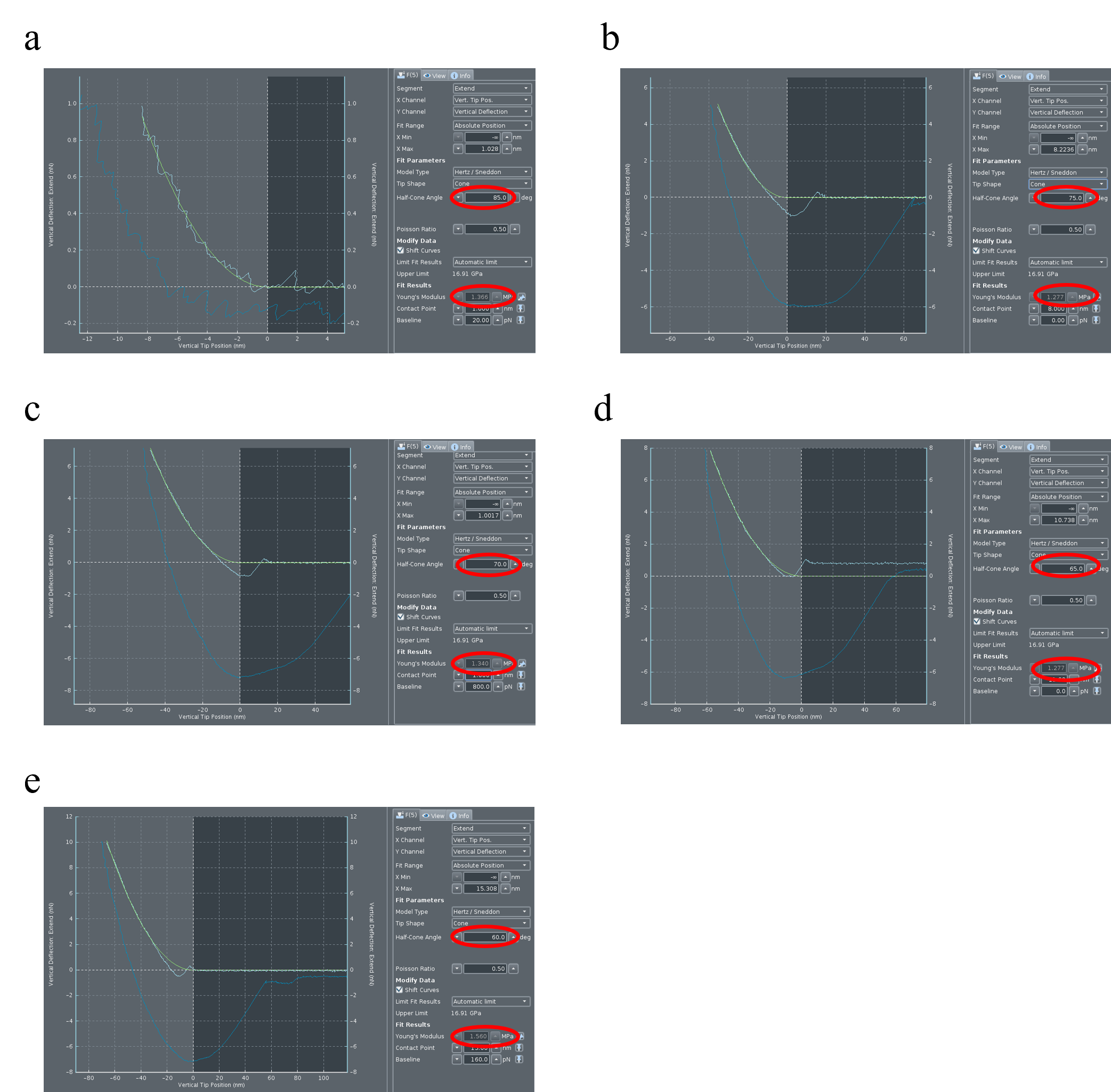  fit  fit  adhesion  elastic modulus  Supplementary Figure S4 Representative raw data analysis of PDMS moduli at sub-100 nm indentations. F-D curve conical angle fittings on PDMS. Loading forces of 1, 5, 7 and 10 nN were used to indent PDMS to progressively increasing depths and the conical half-angles were adjusted to maintain an elastic modulus ~ 1.3 MPa (circled in red in each image). Annotated curves (white arrows) depicting the region of the F-D curve used for (a) calculation of elastic modulus (b) shows the adhesion energy which is disregarded in the Hertzian models and known to lead to slight overestimation of elastic modulus. Indentation depths were (a) 8 nm (b) 36 nm (c) 48 nm (d) 58 nm (e) 67 nm. To check for any ambiguity with actual indentation contact points the same F-D curves were routinely checked at different contact points to adjust the slope fitting (visible as the light grey background area which contrasts against the unfitted dark grey region in all raw F-D plots); represented in (c, d) (dashed yellow arrows) and the conical half-angle was adjusted to maintain a modulus ~ 1.3 MPa as a result of the concomitant change in indentation depth. Large adhesion forces were unavoidable with increased loading and indentation and represent a limitation in our model. Black arrows in (b,d,e) show the hydrodynamic drag force remains constant at ~ 200 pN at all preselected loading forces. | | |
| \| a \| b \| \| --- \| --- \| \| 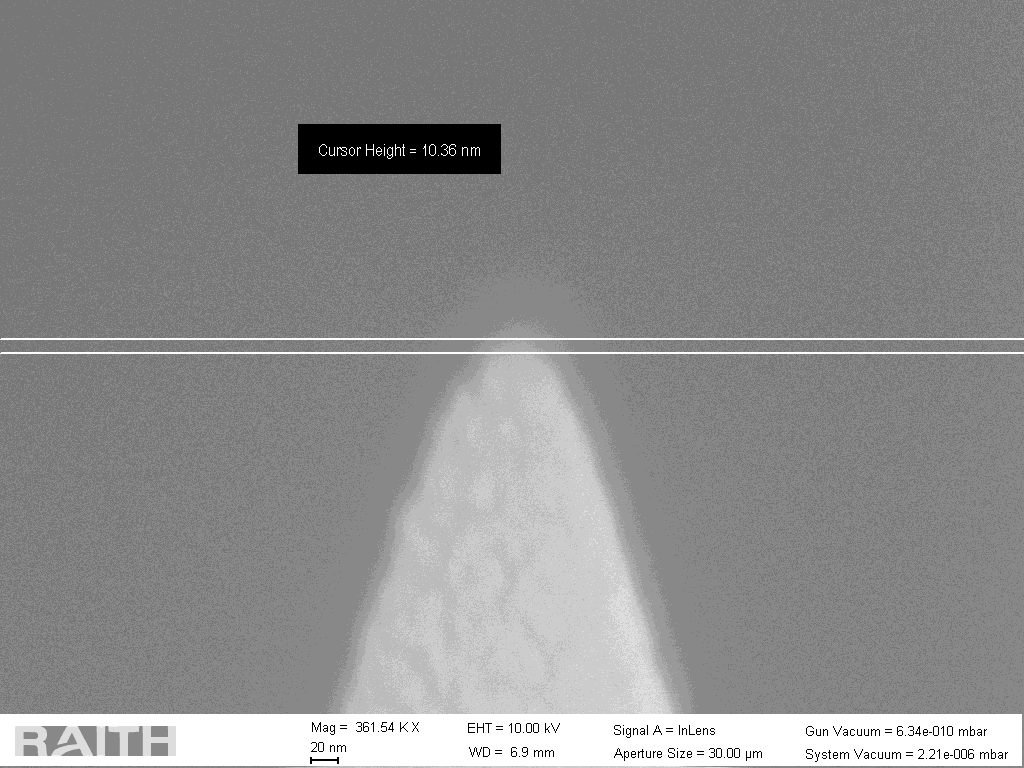 \| 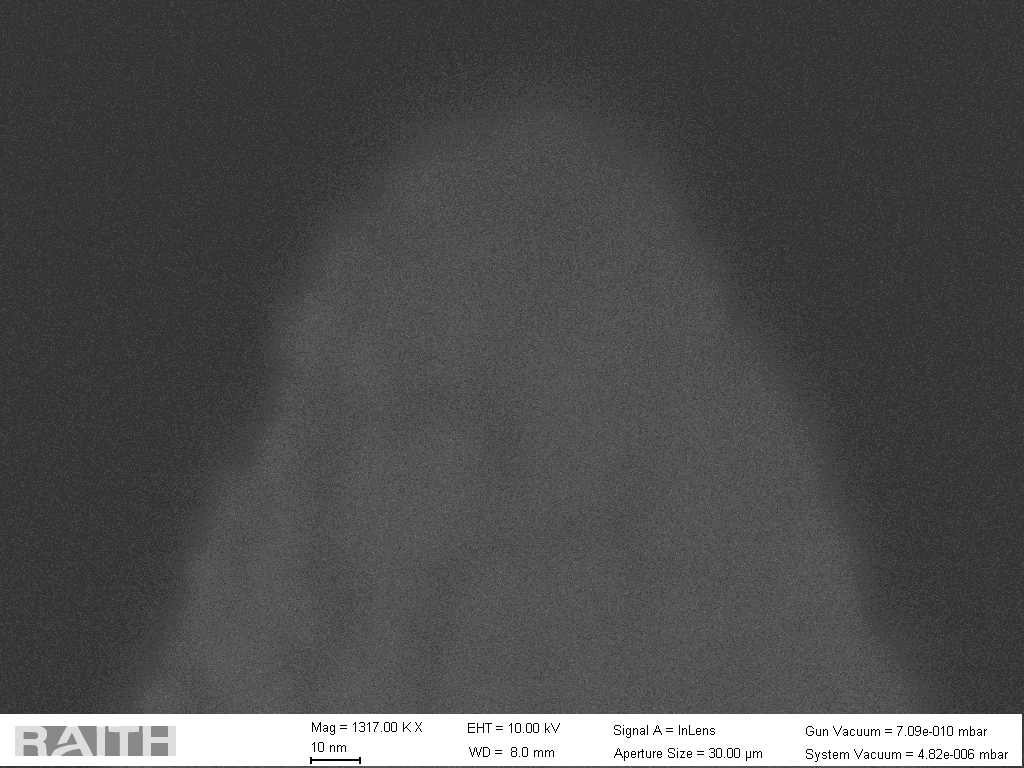 \| | | |

Supplementary Figure S5 Sputter-coated tips show a rough morphology. Initial SEM was performed with a ~ 10 nm layer of gold sputter-coated onto the AFM tip, but the tip was seen to adopt a rough morphology with rounded clumps of coating visible at all length scales. The parallel lines in Figure a denote the top 10 nm from the tip apex and were applied within the manufacturer software.

| a | b | c | |
| --- | --- | --- | --- |
| 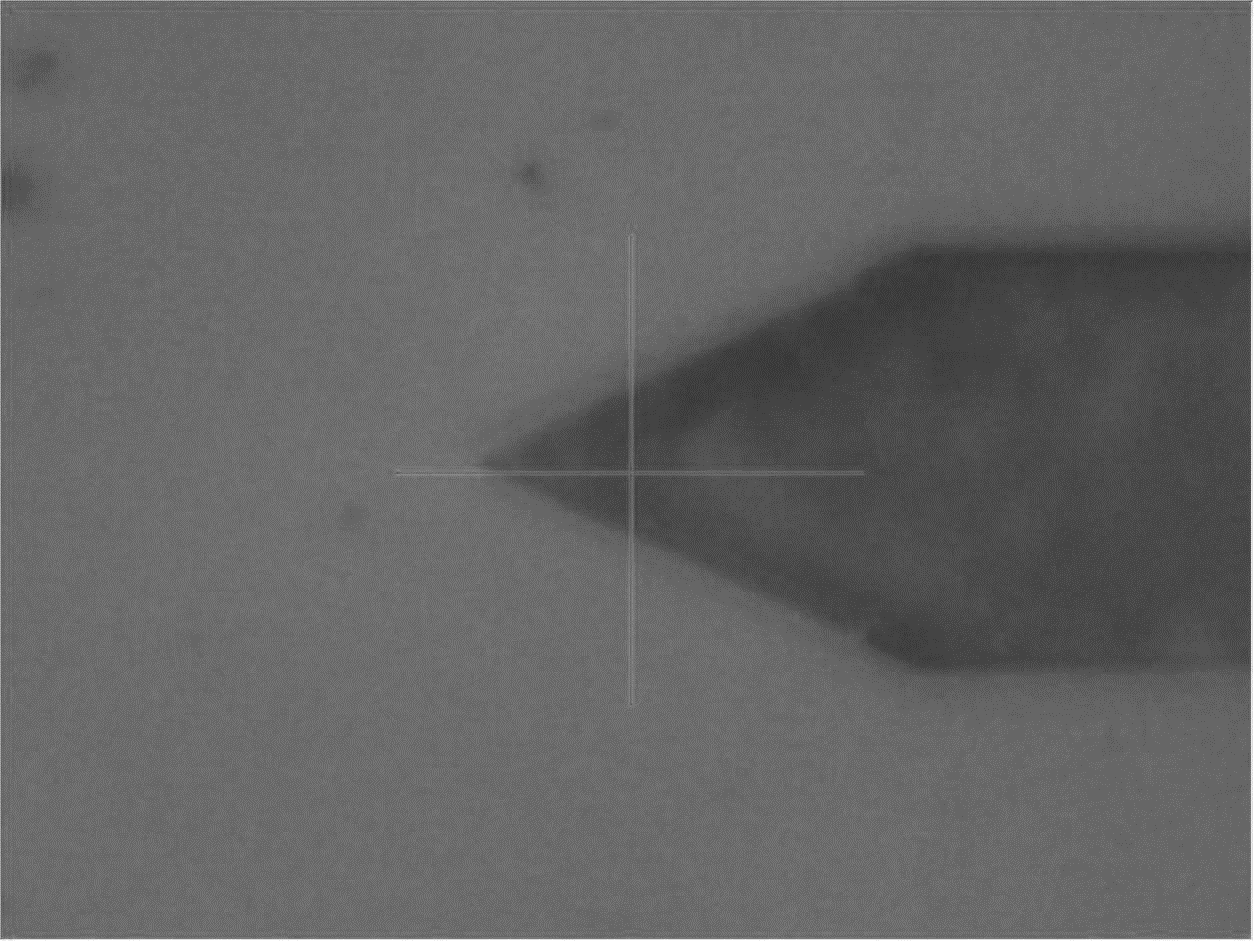 | 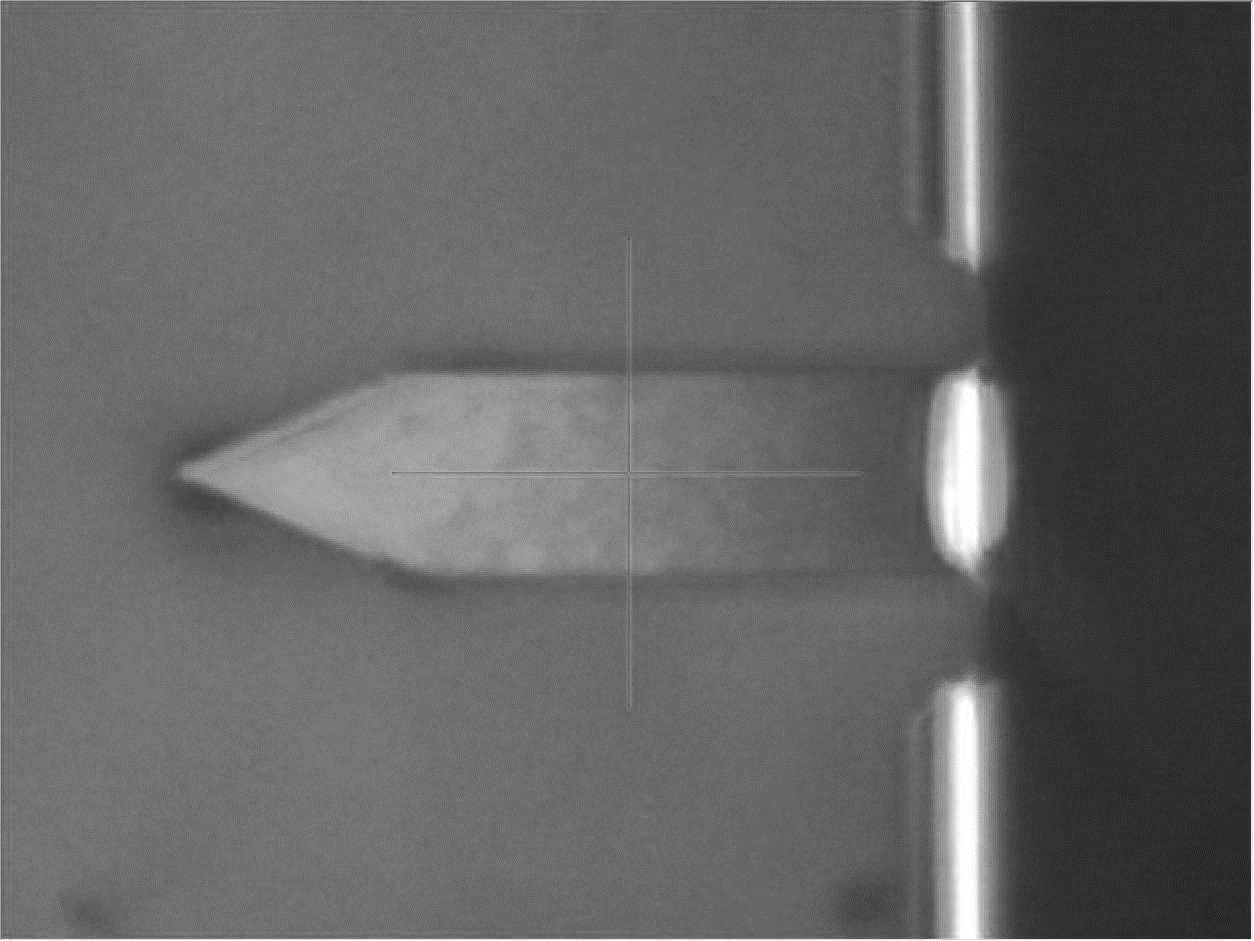 | 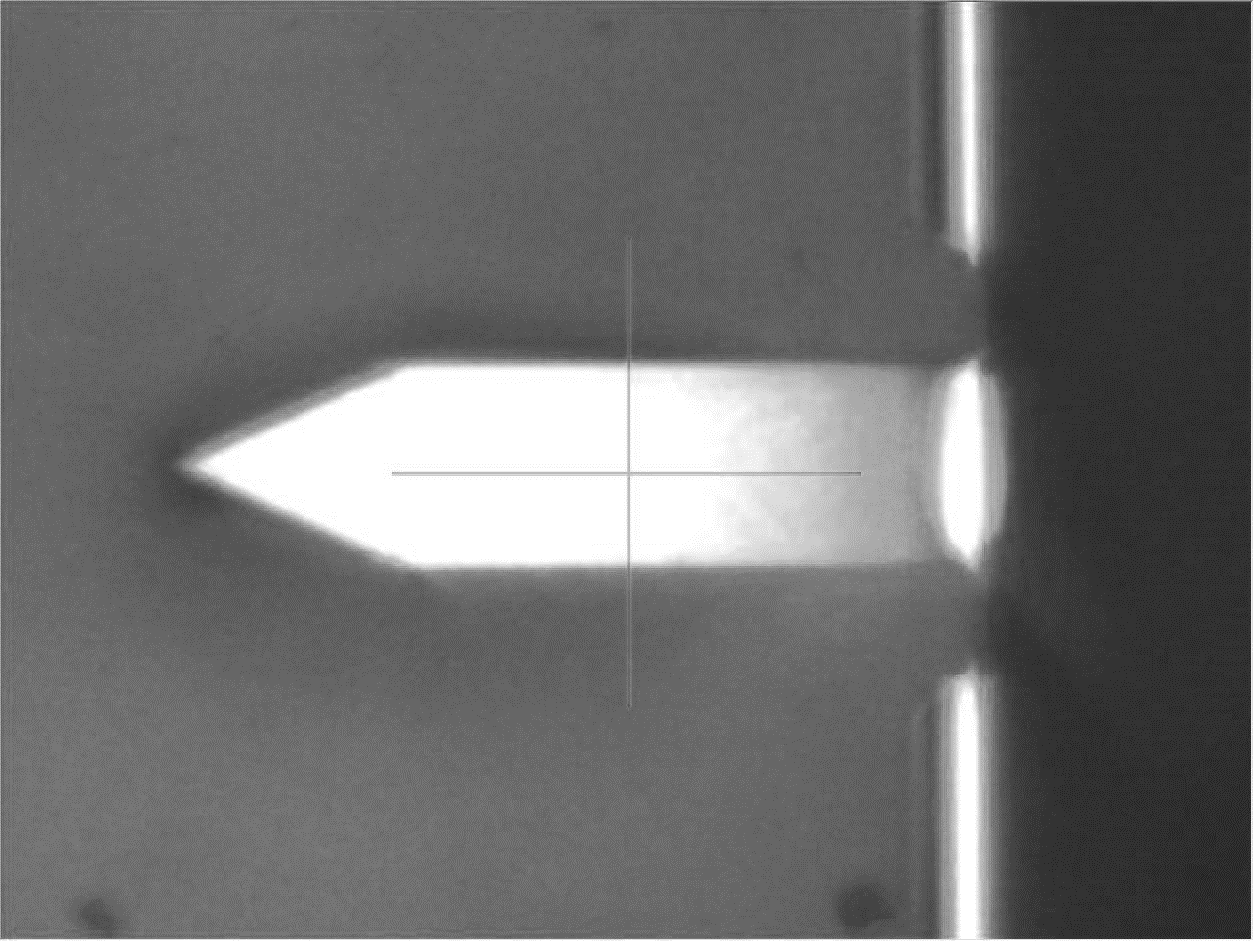 | |
| 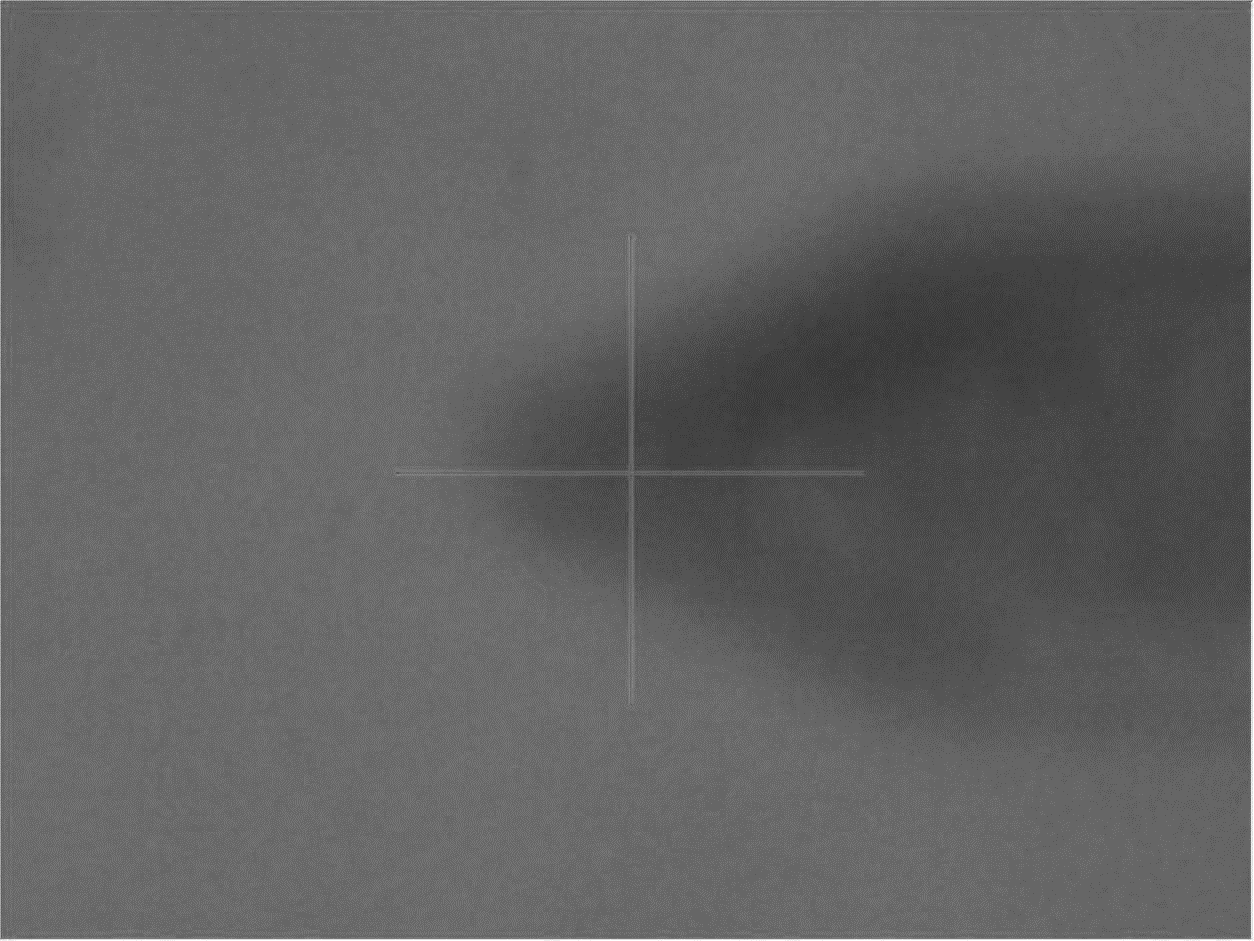 | 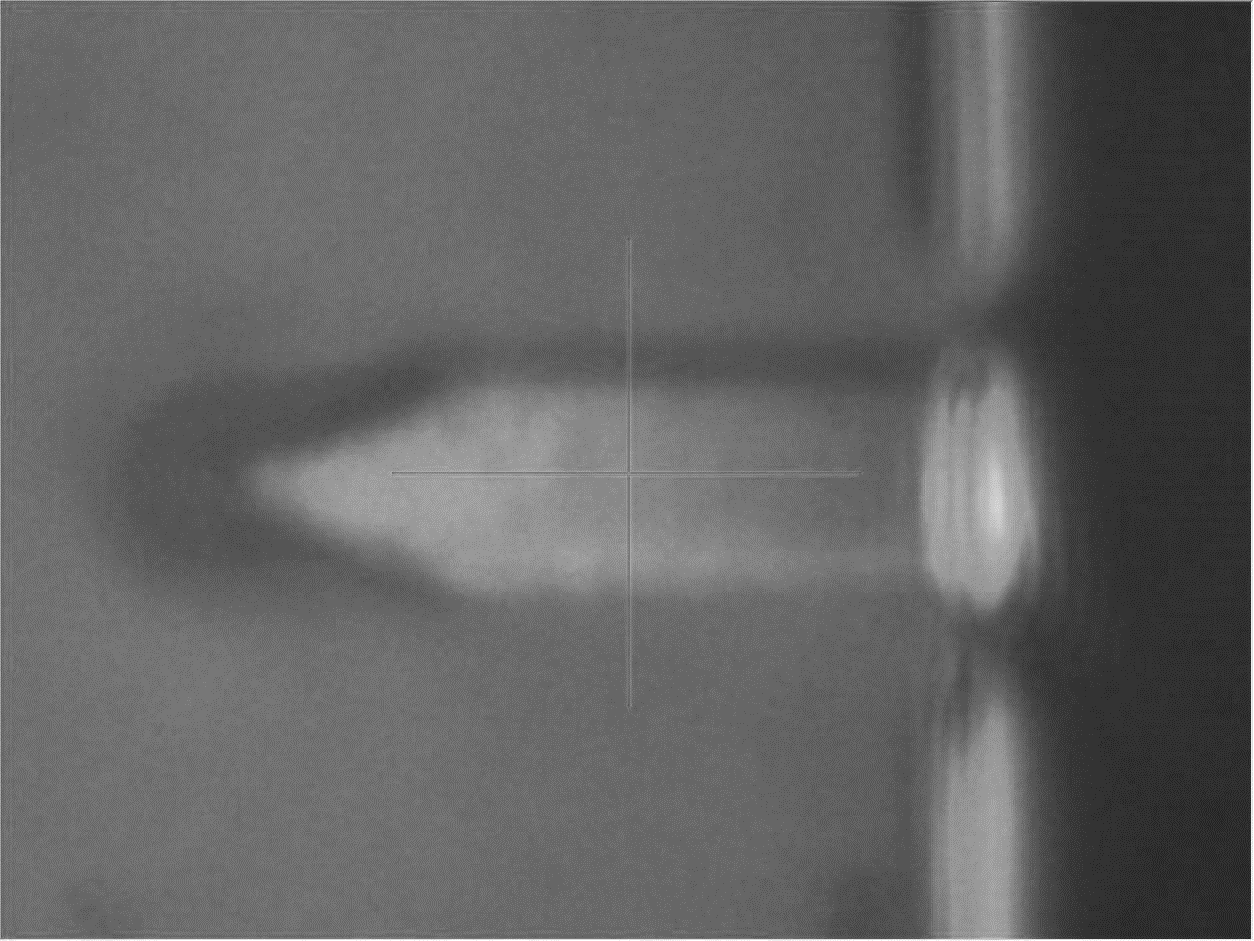 | 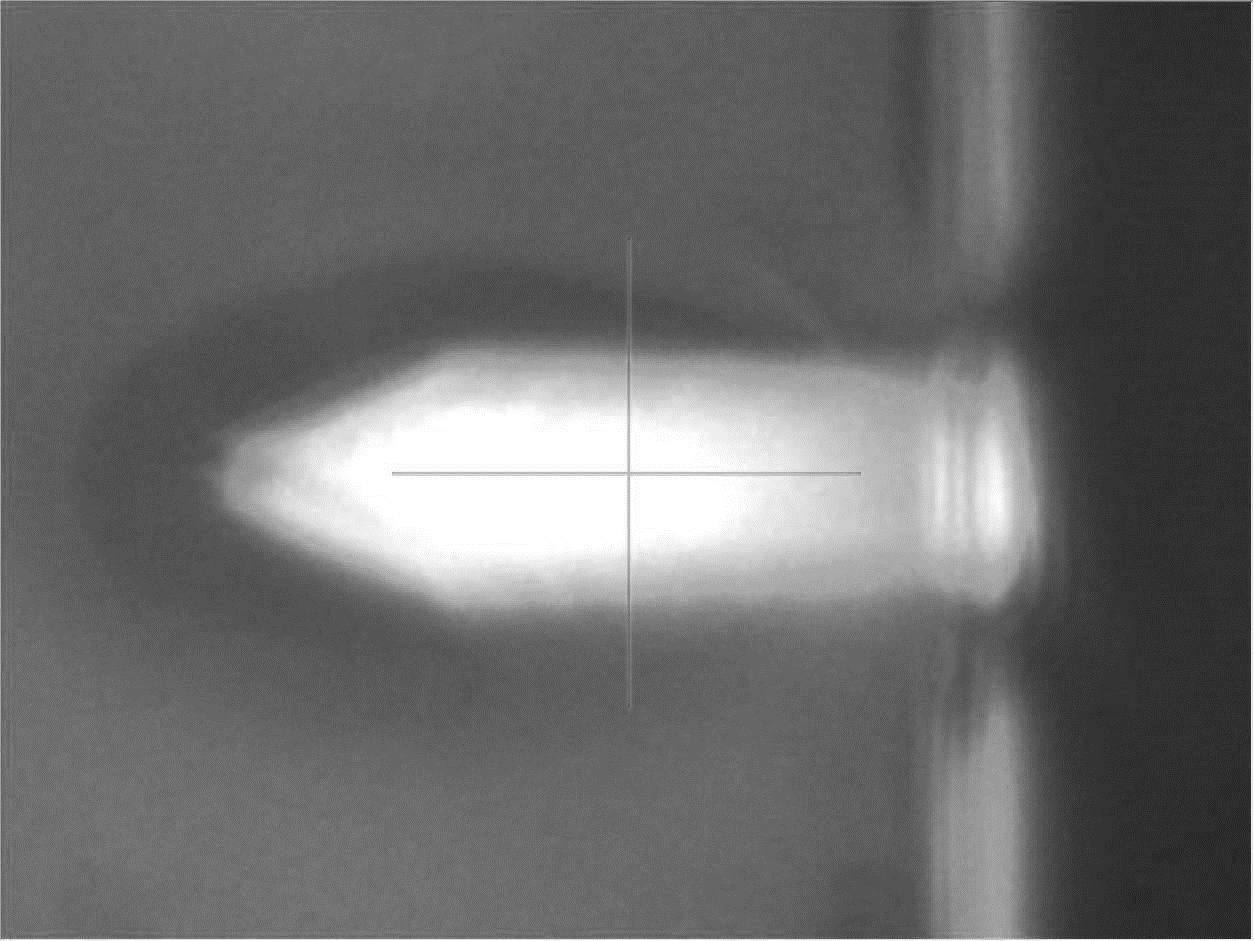 | |
| Supplementary Figure S6 Optical images of AFM probe-induced sink-in on PDMS. Top-down optical micrographs of a tapping probe interacting with 10:1 (w/w) PDMS. The top row shows images focused on the probe and the bottom row shows defocused images centered at the sink-in to better display the indentation area. Dashed blue lines encircle the deformed regions (a) Above the PDMS surface with no contact. (b) Moderate load and deflection of the probe and the concomitant sink-in effect on the PDMS. (c) High load and deflection with greater sink-in deformation continuing along the length of the probe. | | | |

| a | b c |
| --- | --- |
| 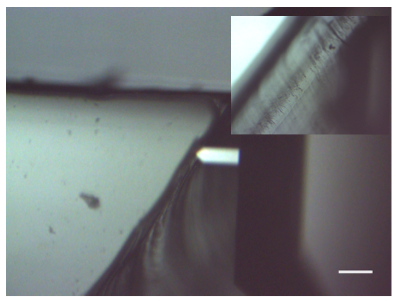 | 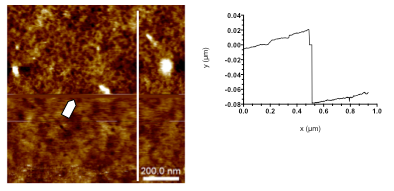 |

Supplementary Figure S7 Analysis of the molecular architecture of PDMS at the surface and sub-surface. (a) A TESPA-V2 cantilever is positioned near a blade-sliced surface and sub-surface interface prior to AFM image capture of 10:1 (w/w) PDMS. Scale bar is 100 nm. Inset (white dotted box) shows a magnified region. (b) Sliced PDMS AFM phase image of surface and sub-surface. The thick line (arrowed) represents the interface, and the AFM was not able to sufficiently capture image data, due to the steep angle. The vertical white line represents a line profile fitted to the AFM height image of the same data. (c) The line profile data report a sub-surface depth ~ 100 nm. Image was 3^rd^ order flattened. Phase angle (dark to light) = - 28.4° to 5.7°. Image size = 1 x 1 µm.

| a | b |
| --- | --- |
| 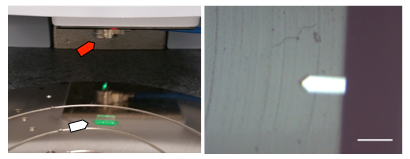 | |

Supplementary Figure S8 Snap-freezing method for obtaining µm and mm sub-surface AFM images. Exposed internal structure of PDMS prior to AFM analysis. PDMS has been briefly immersed for 20 s in liquid nitrogen and cracked to expose a smooth internal surface. (a) The PDMS was secured to the microscope stage (white arrow) and the exposed internal surface was oriented upwards for AFM analysis. The light from the illuminator has created a green reflection from the mounting elastomer. The cantilever is mounted in the *Z* scanner connector (red arrow). (b) An optical micrograph shows a TESPA-V2 cantilever above the internal surface. Multiple concentric lines appear to be evenly distributed throughout. Scale bar = 100 nm.

| a | b | c |
| --- | --- | --- |
| 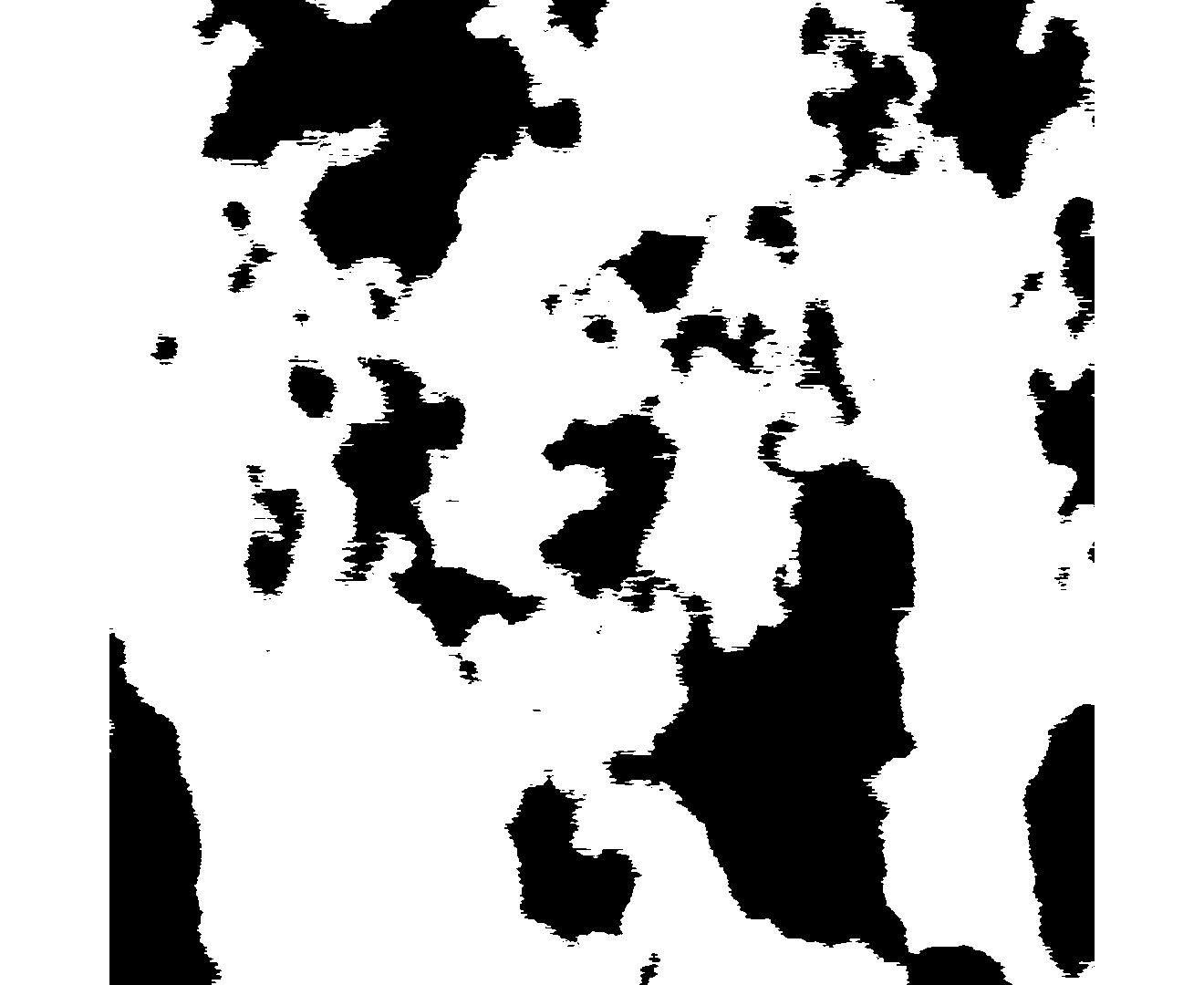 | 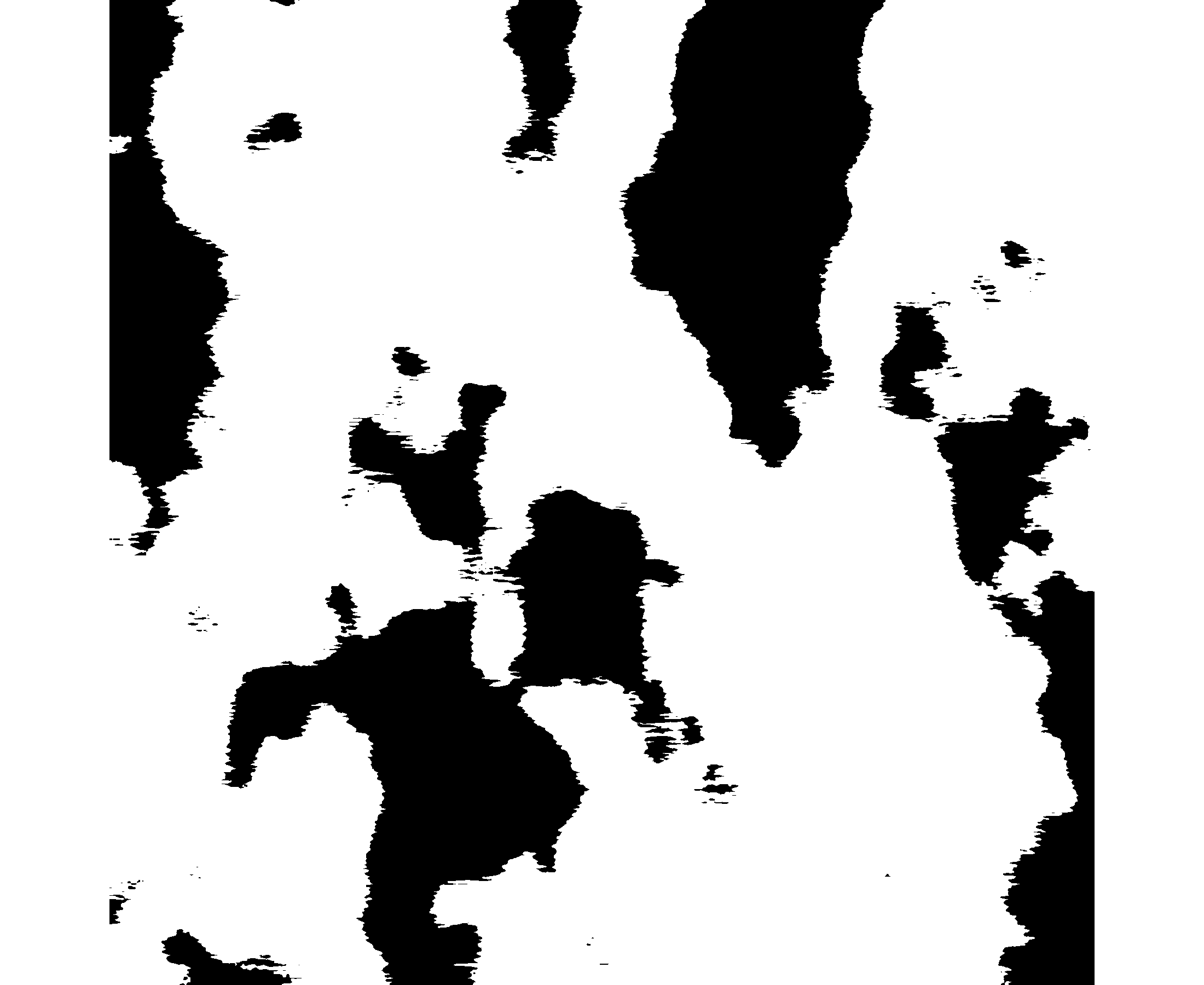 | 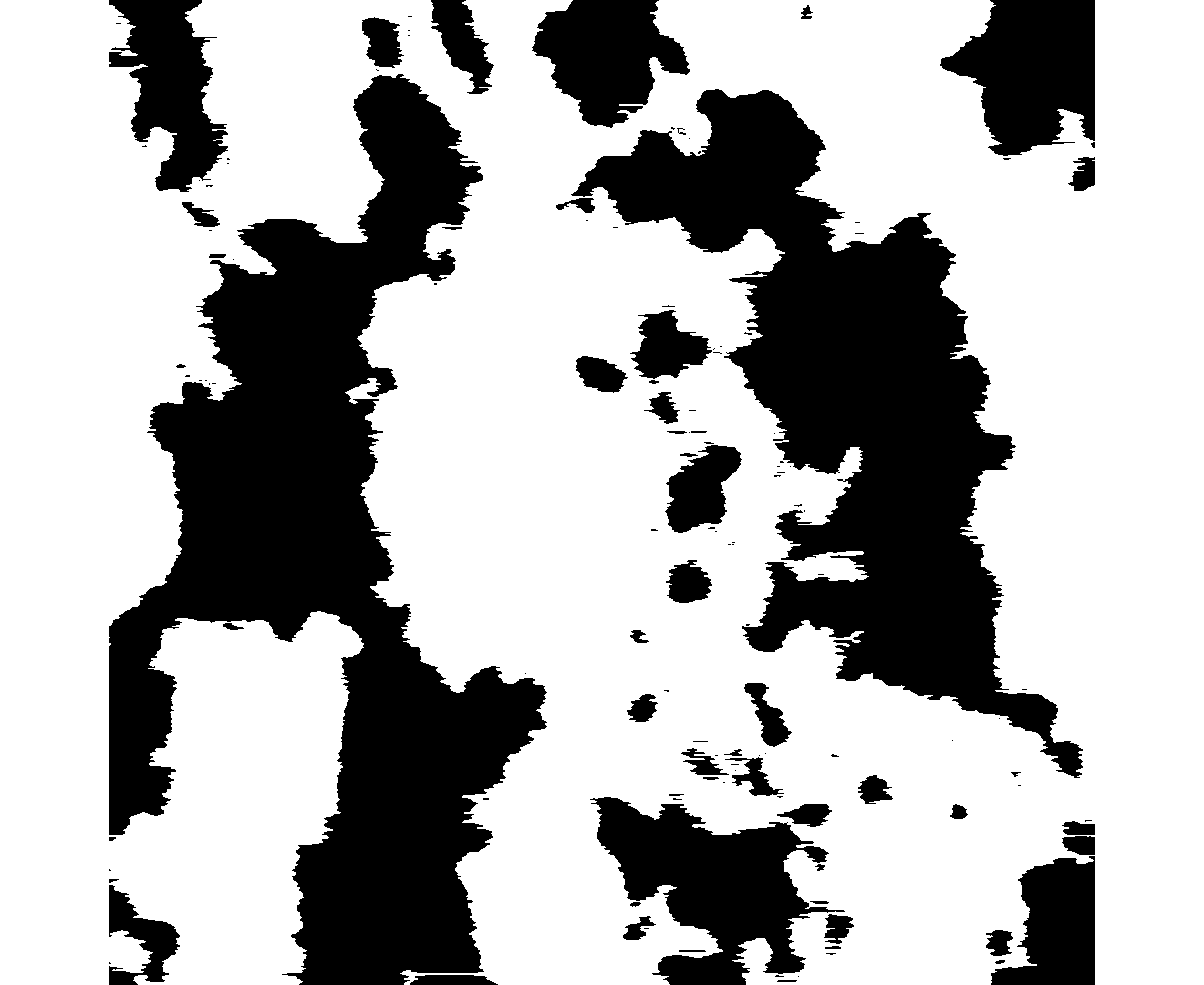 |
| d | e | f |
| 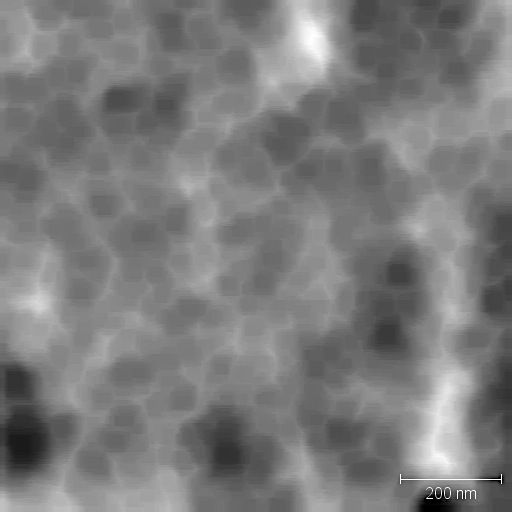 | 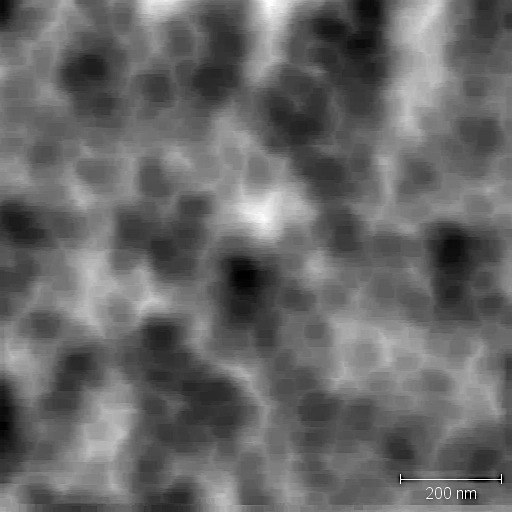 | 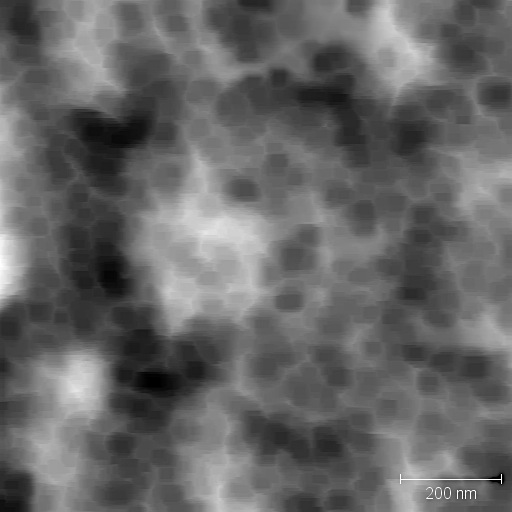 |
| Supplementary Figure S9 Threshold mask and edited AFM images of the PDMS sub-surface. (a-c) Fiji/ImageJ Analyze particles - mask outputs used to estimate the porosity of the sub-surface scans i, ii, and iii respectively (from Figure 3c). (d-e) To reveal more structural details the same AFM images used for the above processing have been binary filtered within Fiji/ImageJ to remove pixels from the edges of black objects with 8, 10 and 8 pixel sizes respectively. *Z* scale = (d) 32.6 nm (e) 20.5 nm (f) 57.7 nm. Phase angle (dark to light) = (d) 26.4° to 34.0° (e) -21.4º to 31.4º (f) 29.7º to 41.7º. Image pixel density = 512 x 512. All images are 1 x 1 µm scan size. | | |

| a | b | c | |
| --- | --- | --- | --- |
| 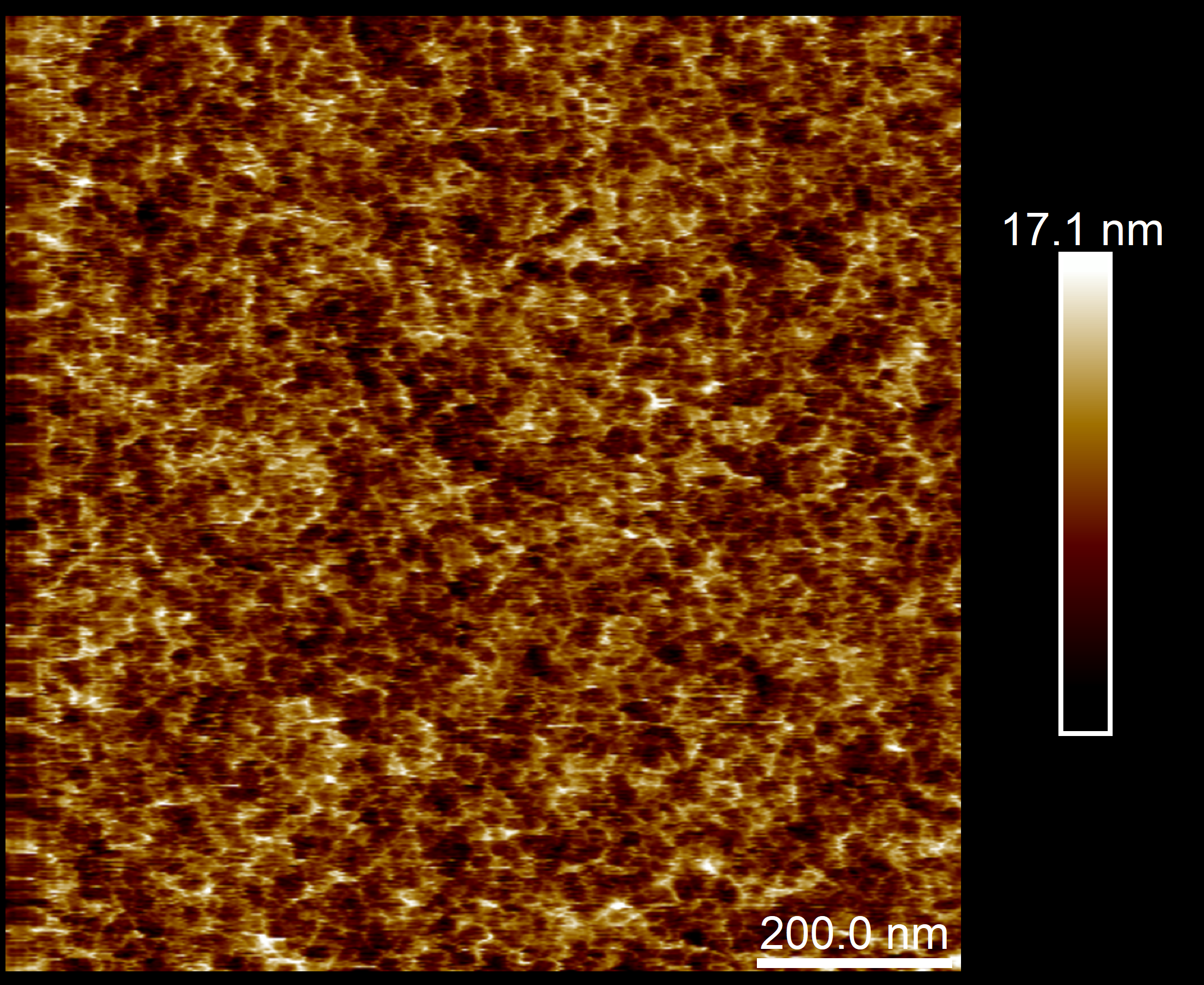 | 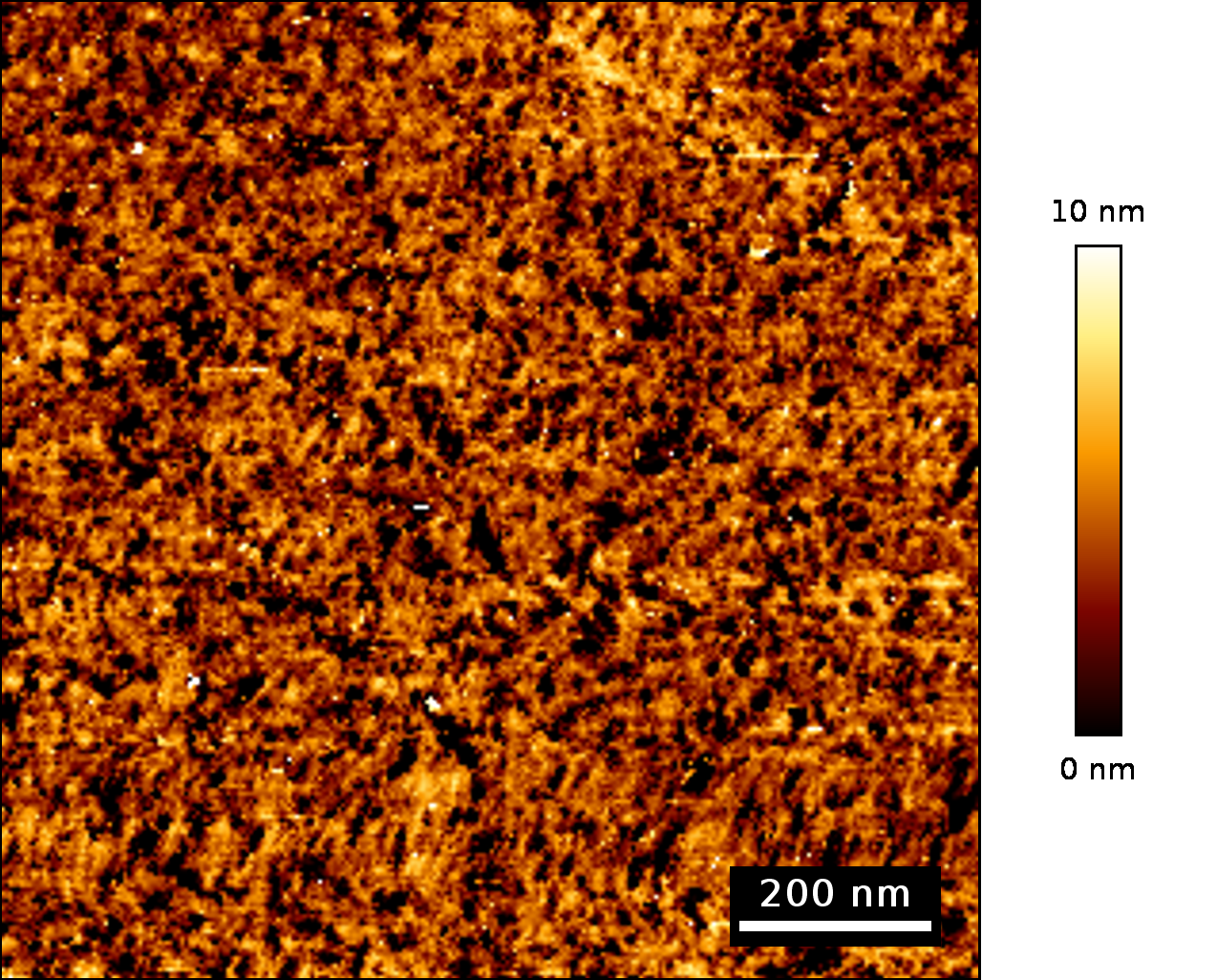 | 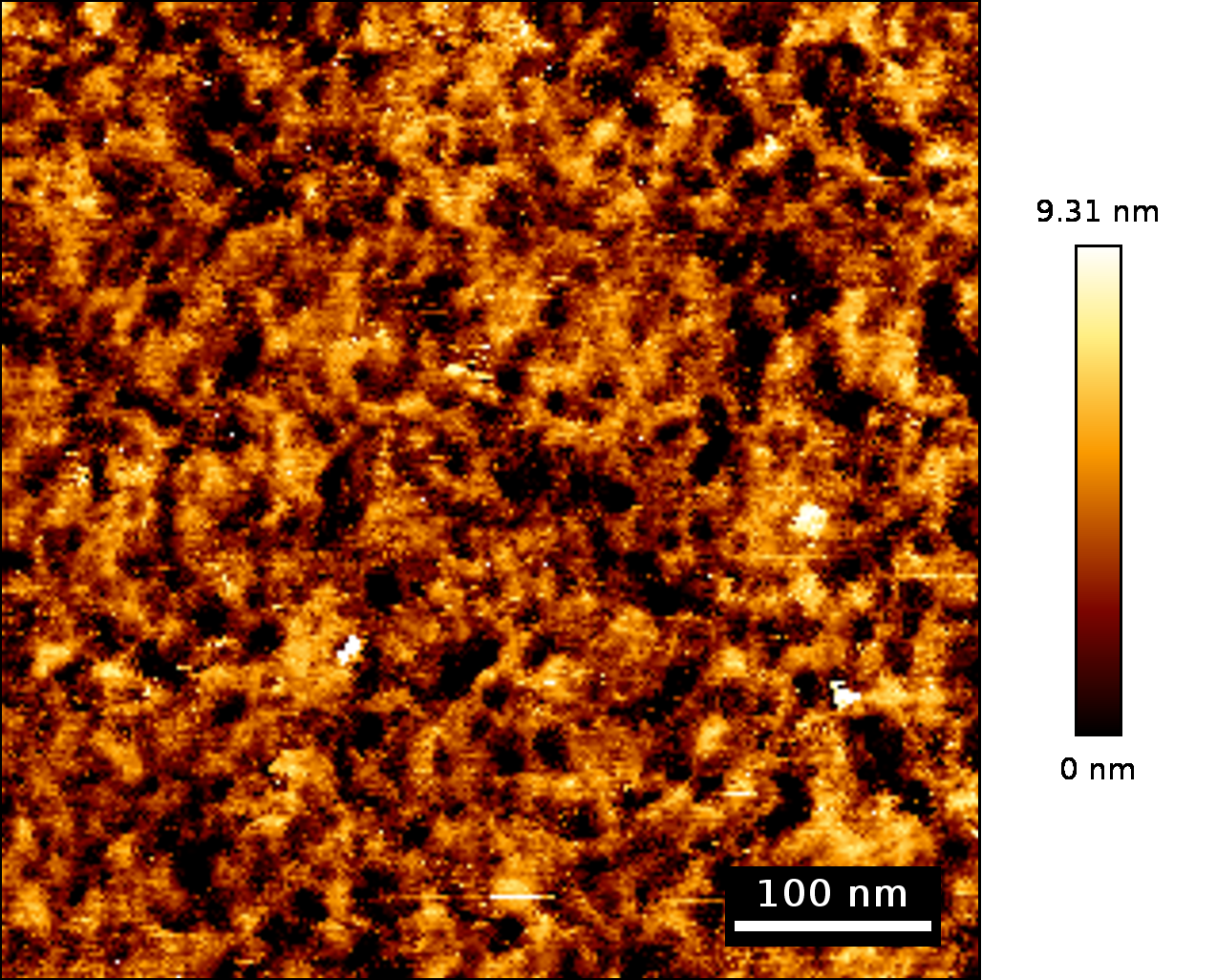 | |
| Supplementary Figure S10 PDMS architecture from alternative AFMs and conditions. Comparisons against the molecular architecture of PDMS observed using the Bruker FastScan in air (Figure 3 and 4 and S7b). (a) 1 x 1 µm topograph using a Dimension 3100 (Veeco) AFM in intermittent contact mode, with TESPA-V2 (Bruker) cantilevers in air. (b,c) 1 x 1 µm and 0.5 x 0.5 µm topographs, respectively, in deionized water using a Biolever mini (Olympus) probe in QI™ mode on an Ultraspeed AFM (JPK). | | | |

**Supporting Information References**

Ebenstein, D. M., & Pruitt, L. A. (2004). Nanoindentation of soft hydrated materials for application to vascular tissues. *Journal of Biomedical Materials Research*, *69A*(2), 222–232. https://doi.org/10.1002/jbm.a.20096

Oliver, W. C., & Pharr, G. M. (1992). An improved technique for determining hardness and elastic modulus using load and displacement sensing indentation experiments. *Journal of Materials Research*, *7*(6), 1564.
